# Injectable *in-situ* curable hydrogel for medullary cavity hemostasis

**DOI:** 10.3389/fbioe.2025.1658768

**Published:** 2025-09-23

**Authors:** Xiangxiao Meng, Fei Liu, Chao Shi, Yichun Dou, Jingshuang Zhang, Xieyuan Jiang, Rui Shi

**Affiliations:** ^1^ State Key Laboratory of Organic-Inorganic Composites, College of Materials Science and Engineering, Beijing University of Chemical Technology, Beijing, China; ^2^ Beijing Research Institute of Traumatology and Orthopaedics, Beijing Jishuitan Hospital, Capital Medical University, Beijing, China; ^3^ Research Center for Intelligent Design and Manufacturing of High-End Oil and Gas Equipment, College of Mechanical and Transportation Engineering, China University of Petroleum, Beijing, China

**Keywords:** non-compression hemostasis, hemostatic gel, thrombin, *in situ* curing, degradable

## Abstract

Achieving hemostasis in the narrow and elongated medullary cavity is a challenge in bone hemostasis. This study developed an *in-situ* hemostatic hydrogel based on the Schiff base reaction principle, which was cross-linked by the aldehyde groups of dialdehyde-functionalized PEG (DF-PEG) and the amino groups of carboxymethyl chitosan/thrombin (CMCS/Th). We utilized the injectability and rapid curing capability of the hydrogel to achieve hemostasis through physical occlusion of the wound. Gel10 possessed an interpenetrating porous structure and cationic groups, with a water absorption capacity of approximately 269%. The resulting Gel10 can promote the aggregation of blood cells by adsorbing blood. Furthermore, Gel10 was successfully modified with thrombin to create Gel10-Th200, which enhanced mechanical properties and promoted secondary hemostasis and bone tissue repair. In a rabbit model of medullary cavity hemorrhage, compared with the control group (medical gauze) which required external pressure, Gel10-Th200 showed remarkable performance. It significantly shortened the bleeding time and reduced blood loss. Moreover, thanks to its enhanced *in situ* hardness, Gel10-Th200 did not require external compression. Additionally, Gel10-Th200 exhibited excellent cell compatibility and negligible hemolytic effects. In summary, Gel10-Th200, with its favorable shape adaptability and rapid curing ability, is considered a promising option for hemostasis in this complex bleeding site within the medullary cavity.

## 1 Introduction

With the intensification of population aging, the incidence of fractures in the elderly is gradually increasing. Intramedullary nail is an effective means of treating fractures, but during intramedullary nail implantation surgery, a long and narrow medullary cavity channel is formed, causing uncontrollable bleeding. Hemostasis is a complex process that typically involves two steps. The first step is primary hemostasis, where platelets and chemical substances congregate at the site of bleeding (Li et al., 2023; Clemetson, 2012), further enhancing vascular constriction. The second step is secondary hemostasis, which entails the activation of the coagulation cascade (Lippi and Favaloro et al., 2018), leading to the transformation of fibrinogen into fibrin and eventually causing the generation of a blood clot. This understanding has inspired the development of various hemostatic materials. Bone wax is currently the most widely used hemostatic agent in clinical practice for bone hemorrhage. However, it is non-absorbable and known to provoke inflammatory reactions as well as interfere with the natural bone healing process. To overcome these limitations, a variety of absorbable hemostatic materials have been developed, including alkylene oxide copolymers (e.g., Ostene^®^), oxidized regenerated cellulose (e.g., Surgicel^®^), gelatin sponges (e.g., Gelfoam^®^), microfibrillar collagen (e.g., Avitene^®^), and collagen-based substrates. While these materials have demonstrated efficacy in controlling superficial bone bleeding, they primarily function through mechanical compression at the bleeding site. This mechanism renders them unsuitable for filling and sealing hemorrhages within the bone marrow cavity, and may additionally contribute to delayed bone union. (Ding et al., 2024; Suchý et al., 2020; Jensen et al., 2009; Zhou et al., 2019; Maxwell et al., 1992). However, these materials either have undesirable side effects or are difficult to use for filling the marrow cavity.

Hydrogels have attracted intense interest in the biomedical field due to their excellent biocompatibility and injectability, and they especially show great potential in hemostasis and tissue repair (Bertsch et al., 2022). The core technical approach to achieving the injectability of hydrogels essentially involves triggering the sol-gel transition process through external stimuli. Current mainstream methods include pH-responsive cross-linking, photoinitiator-induced cross-linking, temperature-sensitive phase transition, and organic cross-linker-induced cross-linking. These techniques share a common characteristic: they all rely on external stimuli (such as pH changes, light exposure, temperature alterations, or chemical reagent addition) to trigger the gelation reaction, which may pose risks of biocompatibility issues or operational complexity (Lavanya et al., 2020; Wang et al., 2025). In contrast, hydrogel systems constructed based on Schiff base dynamic covalent bonds possess a unique advantage in that they can achieve autonomous sol-gel transition without external stimuli. These hydrogels form dynamic network structures through the reversible condensation reaction between aldehyde groups and amino groups. This chemical property endows them with dual characteristics: high biological safety and integrated self-healing injectability (Zhang et al., 2023; Huang et al., 2023). The above characteristics make Schiff base hydrogels an innovative solution for medullary cavity hemostasis. Their autonomous gelation mechanism, which operates without external intervention, can precisely adapt to the complex physiological environment of the medullary cavity, providing a new strategy for traumatic hemostasis that combines safety and functionality. Currently, there are numerous application examples of this type of hydrogel. Zhou designed a three-component dynamic self-healing hydrogel to study the controlled release of carbazole dyes with different concentrations of DF-PEG, and this hydrogel can be applied to cancer treatment and drug delivery systems (Zhou et al., 2018). Zhang simply mixed the synthesized agarose−ethylenediamine conjugate (AG-NH_2_) and DF-PEG solutions to prepare a pH-responsive AG-NH2/DF-PEG hydrogels (AD hydrogels) that can be used as a wound dressing (Zhang et al., 2018). However, the existing injectable hydrogel technology still has limitations: its injection process relies on the physical pushing of the gel state, which is restricted by the complex three-dimensional structure of the medullary cavity and difficult to achieve complete filling of irregular cavities. At the same time, the kinetic process of the self-healing mechanism is relatively slow, ultimately affecting the overall hemostatic efficiency. To achieve optimal clinical outcomes by reducing procedure time, bacterial infiltration, and tissue inflammation, an ideal osseous hemostatic hydrogel must exhibit rapid *in situ* gelation, high fluidity, and multifunctional capabilities (Li et al., 2024). Wang prepared an *in-situ* cured CMCS/DF-PEG hydrogel, but its curing time and mechanical properties need to be improved to achieve medullary hemostasis. Currently, this hydrogel is used for intraperitoneal anti-adhesion (Wang et al., 2023). Based on the above research, this paper designed a fast-curing and well-blocked hydrogel. This hydrogel contains chitosan components that can attract anionic blood cells (Lunkov et al., 2023; Yan et al., 2022), promote their aggregation, and initiate primary hemostasis. As a hemostatic gel, its drug-loading properties can also be used to load bioactive hemostatic factors, thereby stimulating secondary hemostasis.

Agents for hemostasis, like batroxobin, thrombin, and fibrinogen, play a role in promoting the clustering of blood cells at the site of the wound. Thrombin, which can be used in various combinations, is the most widely used (Larsen and Hvas, 2021). For example, thrombin can be loaded onto hemostatic starch or hemostatic sponges, significantly enhancing their hemostatic effectiveness through their synergistic action (Li et al., 2019; Li et al., 2018; Zhang Y. et al., 2024). However, none of these methods is suitable for addressing bleeding in narrow or irregular medullary cavities, resulting in reduced hemostatic efficiency. Moreover, injectable hydrogels loaded with thrombin can effectively integrate the coagulation cascade with bioactive properties to achieve hemostasis in complex wounds (Guo et al., 2023; Zhang B. et al., 2024; Zhou et al., 2023). Thrombin also has multiple roles. In hemostasis, it can promote the conversion of fibrinogen to fibrin. In non-hemostasis, by activating PARs, thrombin can regulate physiological processes such as embryonic development and wound healing (Posma et al., 2016).

In this study, our objective was to fabricate a cross-linked composite made up of CMCS, DF-PEG, and thrombin. We intended to use this injectable *in-situ* curing hydrogel for hemorrhage control. Specifically, we adjusted the cross-linking ratio of CMCS and DF-PEG to achieve an optimal curing speed and sealing ability for the hydrogel. The hydrogel’s porous structure enabled substantial blood absorption. Meanwhile, the cationic groups within CMCS participated in electrostatic interactions with anionic blood cells, thereby facilitating their aggregation. The incorporation of thrombin served a dual purpose: on one hand, it improved the mechanical characteristics of the hydrogel *via* cross-linking reactions; on the other hand, it efficiently promoted the transformation of fibrinogen. Based on the physicochemical properties of this composite material, we assessed its potential as a local hemostatic agent. Additionally, we evaluated the material’s safety and biocompatibility through CCK8 assays and hemolysis tests. Moreover, we observed how blood cells clumped together and how fibrin was formed to figure out the hemostatic mechanism of the material. Finally, by applying the hydrogel to a medullary canal hemorrhage model in rabbits, we confirmed its hemostatic effect and its role in bone tissue repair.

## 2 Methods

### 2.1 Materials

DF-PEG (with a molecular weight approximately equal to 2000 Da) was procured from Tanshtech (Guangzhou, China). CMCS (having a carboxylation degree exceeding 80%) was obtained from Yuan ye Biotechnology Co., Ltd. (Shanghai, China). Thrombin was purchased from Biorigin (Beijing, China). Deionized water was employed throughout this study and all other chemicals utilized were of analytical grade.

### 2.2 Preparation

This system was formed by the cross-linking of three components: DF-PEG, CMCS, and thrombin (Th), the detailed recipe is shown in Table 1. The specific synthesis route was as follows: first, the drug-free hydrogel (Gel) was screened. The Gel was formed *via* Schiff base cross-linking between the aldehyde groups of DF-PEG and the amino groups of CMCS. It was prepared by mixing DF-PEG (15% w/v) with CMCS at four different concentrations (6%, 8%, 10%, and 12% w/v) in a 1:1 (v:v) ratio, resulting in four types of drug-free hydrogels (Gel6, Gel8, Gel10, Gel12). Through *in vitro* physicochemical property characterization, Gel10 was selected as the optimal formulation. Subsequently, thrombin-loaded hydrogels (Gel-Th) were prepared using Gel10 as the carrier. The Gel-Th was formed through further Schiff base cross-linking between the aldehyde groups of DF-PEG and the amino groups of both CMCS and thrombin. Thrombin was dissolved in deionized water to prepare a solution with a concentration of 1000 U/mL. Four different volumes of this thrombin solution (250 μL, 500 μL, 750 μL, and 1,000 μL) were added respectively to vials containing 0.5 g of CMCS. Deionized water was added to prepare 5 mL of precursor solution 1. Meanwhile, DF-PEG was prepared into precursor solution 2 at a concentration of 15% (w/v). The two precursor solutions were mixed at a 1:1 volume ratio to prepare four types of drug-loaded hydrogels (Gel10-Th50, Gel10-Th100, Gel10-Th150, and Gel10-Th200). The optimal ratio of Gel10-Th200 was then determined through subsequent biological tests.

**TABLE 1:** Formulation codes chemical composition.

Formulation codes	Chemical composition
Gel 6	DF-PEG 15%/CMCS 6% (w/v)
Gel 8	DF-PEG 15%/CMCS 8% (w/v)
Gel 10	DF-PEG 15%/CMCS 10% (w/v)
Gel 12	DF-PEG 15%/CMCS 12% (w/v)
Gel10-Th50	DF-PEG 15%/CMCS 10% (w/v), Th50U/mL
Gel10-Th100	DF-PEG 15%/CMCS 10% (w/v), Th100U/mL
Gel10-Th150	DF-PEG 15%/CMCS 10% (w/v), Th150U/mL
Gel10-Th200	DF-PEG 15%/CMCS 10% (w/v), Th200U/mL

### 2.3 Characterization

The Fourier transform infrared (FTIR) spectra of DF-PEG, CMCS, CMCS-Th, Gel and Gel-Th were acquired using a Bruker Tensor-37 Fourier Transform Infrared Spectrum Analyzer.

The Energy dispersive spectroscopy (EDS) of Gel and Gel-Th were acquired using Scanning electron microscopy.

### 2.4 Physical property tests of gel hydrogel

#### 2.4.1 Adhesion

The adhesion capability between the hydrogel and the bone tissue was assessed through qualitative tests. The hydrogel was injected and cured onto prepared bone samples, which were then subjected to continuous water flushing to observe whether the hydrogel detached. For further quantitative testing, two pig skin specimens were fixed on the test platform. The hydrogel was evenly applied to the surfaces of the two pig skin specimens and then pressed for 3 min. After that, the plates were separated at a constant speed of 13.4 mm/min. The adhesion of the hydrogel was defined as the maximum load measured during the separation process.

#### 2.4.2 Hydrophilicity

The contact angle was determined using a contact angle goniometer manufactured in Shanghai, China. Deionized water was dropped at three distinct locations on the surface of the sample at room temperature, and the contact angle was photographed and recorded immediately after the liquid was dropped.

#### 2.4.3 Swelling

Cylindrical specimens with a diameter of 1 cm and a height of 5 mm were made. To start with, the samples were weighed ( $W_{0}$ ). After that, the samples were put into deionized water. At predetermined time intervals (2 h,4 h, 6 h), the samples were removed, and the surfaces were gently dried. The weight of the samples was measured again ( $W_{t}$ ), and the swelling rate (%) was evaluated according to Equation 1:

$$\text{Swelling rate }\left(\%\right)=\frac{W_{t}‐W_{0}}{W_{0}}\times 100\%$$ (1)

#### 2.4.4 Liquid sealing

Acrylic glass tubes with a length of 2.00 m and an inner diameter of 3 mm were filled with water to a level of 1.91 m. This water-filling height was equivalent to a systolic blood pressure of 18.68 kPa (140 mmHg) (Brückner et al., 2016). The tubes were sealed with Gel samples measuring 3 cm (n = 3). Subsequently, these fabricated structures were continuously observed until they experienced failure.

#### 2.4.5 Gelation time

The vial tilt method was employed to measure the gelation time at 37 °C. Identical volumes of DF-PEG and CMCS solutions at diverse concentrations were combined in a vial. The time when no liquid flow was observed in the tilted vial was recorded as the gelation time (n = 3).

#### 2.4.6 Micromorphology and pore size analysis

Scanning electron microscopy (SEM, SU8600, Japan) was used to analyze the morphologies of the Gel samples (voltage: 5 kv, amplification factors: 100× and 200×) and the Gel-Th samples (voltage: 5 kv, amplification factors: 100×). Using Nano Measure and Origin software, the pore size of the Gel was quantified based on the SEM images.

#### 2.4.7 Thermal stability analysis

For TGA-DTG, the temperature was set to range from room temperature to 700 °C with a heating rate of 10 °C/min. For DSC, the temperature program was designed as follows: first cooling from room temperature to −20 °C, then heating from −20 °C to 200 °C, with a heating rate of 10 °C/min.

### 2.5 Rheological measurement

Rheological measurement of Gel-Th samples was performed using HAAKE MARS RS6000 rheometer (United States) with a parallel plate at 37 °C in vibration mode. To verify the *in-situ* curing time of the hydrogel, the following operations were carried out: a volume of 0.2 mL of precursor solution 1 (either CMCS solution or CMCS-thrombin solution) was spread onto the surface of the parallel plate (diameter: 20 mm). Subsequently, 0.2 mL of precursor solution 2 (DF-PEG solution) was dropwise added onto the surface of precursor solution 1. We measured storage modulus ( $G^{\prime }$ ) and loss modulus ( $G^{\prime \prime }$ ) as functions of time. In order to verify the influence of stress on the hydrogel, the following operations were carried out: Gel and Gel-Th were made into 2 -mm-thick films, which were then placed on the testing platform of a rheometer. With a fixed frequency of 1 Hz and a stress range from 10^0^ Pa to 10^5^ Pa, the change of modulus with stress was tested.

### 2.6 *In vitro* hemostatic evaluation and hemostasis mechanism

This part of the test was performed using the Gel group and the Gel-Th group as samples.

#### 2.6.1 Blood clotting index (BCI) test

The samples were adjusted to suitable dimensions and positioned at the bottom of the orifice plate. A volume of 20 µL of 0.1 M CaCl2 and 200 µL of anticoagulated whole blood were introduced into a centrifuge tube and then vigorously mixed. Subsequently, the samples were subjected to incubation at 37 °C for 3, 10, 20, 30, and 40 min respectively. Upon completion of the incubation period, we added 1 mL of deionized water. The hemoglobin content was quantified using a UV- visible spectrophotometer (Varioskan Flash, Thermo Scientific, United States) at a wavelength of 545 nm. For the blank control, 10 µL of calcium-fortified anticoagulated whole blood was mixed with 1 mL of deionized water. Every sample underwent an evaluation in three independent replicates. The blood clotting index (%), as defined in the context of this study, was computed in accordance with Equation 2:

$$\text{Blood clotting index }\left(\%\right)=\frac{I_{a}}{I_{w}}\times 100\%$$ (2)

in this context, $I_{a}$ represents the absorbance of the samples, while $I_{w}$ denotes the absorbance of the blank.

#### 2.6.2 Hemolysis test

The whole blood was centrifuged at 1,000 rpm for 10 min to obtain erythrocytes. The samples were then immersed in 1 mL of phosphate-buffered saline (PBS). 20 μL of erythrocytes were introduced into the PBS-soaked samples. The resultant mixture was then incubated at 37 °C for 1 h. Following the incubation, the mixture was spun down at 2,000 rpm for 10 min to acquire the supernatant. The absorbance of this supernatant was determined at 545 nm using a UV-visible spectrophotometer (Varioskan Flash, Thermo Scientific, United States). As per the method described in reference (Wang Y. et al., 2020), deionized water and PBS were combined with untreated erythrocytes, acting as the positive and negative controls respectively. The hemolysis ratio (%), as defined by the experimental protocol, was calculated according to Equation 3:

$$\text{Hemolysis ratio }\left(\%\right)=\frac{D_{t}‐D_{\text{nc}}}{D_{\text{pc}}‐D_{\text{nc}}}\times 100\%$$ (3)

where $D_{t}$ , $D_{\text{nc}}$ and $D_{\text{pc}}$ are the absorbance of the samples, PBS, and deionized water, respectively.

#### 2.6.3 Quantitative analysis of the adhesion of blood cells

The extent of platelet adhesion was quantitatively assessed through lactate dehydrogenase (LDH) activity measurements following established protocols (Shen et al., 2020). Whole blood specimens were processed by centrifugation at 200 rpm for 20 min to isolate platelet-rich plasma (PRP), as described in previous methodology (Raza et al., 2023). Identically sized test samples were then submerged in the obtained PRP solution. After maintaining the specimens at 37 °C for 45 min, non-bonded platelets were removed through three sequential washes with PBS. To lyse the attached platelets, 0.15 mL of PBS solution containing 1% TritonX-100 was applied to the samples, maintaining 37 °C for 60 min. The resulting LDH enzymatic activity was measured through standardized procedures using a commercial detection kit (Biyuntian, China). Platelet adhesion quantification was performed by recording absorbance values at 490 nm wavelength using spectrophotometric analysis. Each experimental condition underwent triplicate tests with final results representing the arithmetic mean of three independent measurements.

Place the samples in a 24-well plate. Take 10 µL of the prepared citric acid-incubated whole blood and add it to the surface of the samples. Add 10 µL of whole blood to the surface of the well-plate as a positive control. Then, incubate the samples statically at 37 °C for 45 min. After the incubation was completed, wash the samples with PBS to assess the adsorption of red blood cells (RBCs). Lyse the RBCs adhered to the samples in deionized water, and measure the absorbance at 540 nm to determine the RBC adsorption. The formula used as Equation 4:

$$\text{RBC adsorption }\left(\%\right)=\frac{{\text{OD}}_{\text{sample}}}{{\text{OD}}_{\text{blank}}}\times 100\%$$ (4)

#### 2.6.4 Qualitative analysis of the adhesion of blood cells

A volume of 20 µL of whole PRP and blood was separately transferred *via* pipetting onto the central area of the sample surface to analyze the adhesion of platelets and erythrocytes. Subsequently, the PRP and blood were incubated at 37 °C under static conditions for 1 h. Once the incubation process concluded, the surface of the sample was carefully rinsed with physiological saline to eliminate any non-adherent blood cells. Subsequently, a 4% paraformaldehyde solution (Biyuntian, China) was used to fully cover the surface of the samples. The samples were then subjected to an incubation process at room temperature for 10 min to fix the cells. The paraformaldehyde solution was then aspirated, and the samples were gently rinsed with deionized water before being air-dried at room temperature. The morphological features of the blood cells that adhered to the membrane surface were examined *via* a laser microscope (VK-150K, Keyens, Japan), capturing both laser images and two-dimensional images.

#### 2.6.5 Fibrin study

The whole blood was subjected to centrifugation at a rotational speed of 2,000 rpm for 10 min to obtain the platelet-poor plasma (PPP). Samples were cut into uniform sizes and placed in culture dishes. A total of 500 µL of the prepared PPP was combined with 50 µL of a 0.1 M calcium chloride solution for recalcification. Following this, 20 µL of the recalcified plasma was precisely dispensed onto the surface of the test specimens and then incubated at 37 °C for 15–20 min. Upon completion of the incubation, the specimens were irrigated with PBS. Subsequently, they were covered with a 4% paraformaldehyde solution for 10 min. The paraformaldehyde solution was aspirated. Subsequently, the test specimens were gently cleansed with deionized water and then allowed to air dry. Fibrin was stained using Masson’s trichrome stain for observation.

### 2.7 *In vitro* drug release and degradation

#### 2.7.1 Drug release

To evaluate the release profile of Th from Gel-Th, the hydrogels were individually submerged in PBS. At every predetermined time interval, the release medium was gathered and subjected to analysis with BCA kits, adhering to the procedure outlined in the instruction manual (Wang C. et al., 2020). The percentage of Th released (%) was computed via Equation 5:

$$\text{RP }\left(\%\right)=\frac{W_{i}}{W_{\text{total}}}\times 100\%$$ (5)

in this equation, $W_{i}$ represented the quantity of Th present in the released medium, while $W_{\text{total}}$ denoted the overall amount of Th. The release experiment was replicated three times to ensure reliability and reproducibility.

To further confirm the mechanism of drug release, four kinetic model formulas were used for fitting, namely the zero-order kinetic model, Higuchi model, the first-order kinetic model and Ritger-Peppas model. The specific model formulas can be found in the Supplementary Material.

#### 2.7.2 Degradation

The Gel10 and Gel10-Th200 specimens were weighed ( $W_{0}$ ) and then fully immersed in PBS maintained at a temperature of 37 °C. At the predetermined time intervals, the samples were retrieved from the PBS, and the moisture on the surface of the hydrogels was carefully blotted dry. The sample was weighed (Wt), and the mass rate was calculated (%) as Equation 6:

$$\text{Mass rate }\left(\%\right)=\frac{W_{t}-W_{0}}{W_{0}}\times 100\%$$ (6)

### 2.8 Cytotoxicity test

Cytocompatibility evaluation was performed through CCK8 colorimetric analysis with murine dermal fibroblasts (L929). For material extraction, 300 mg of Gel10 and Gel10-Th200 samples were immersed in 3 mL Modified Eagle’s Medium (MEM) for 24 h to prepare extraction solutions. Cellular suspensions containing 6 × 10^3^ cells/well were plated in 100 µL MEM supplemented with 10% fetal bovine serum (FBS). Cell cultures were maintained under standard physiological conditions (37 °C, 5% CO_2_) for 24 h prior to treatment. Test groups were administered with graded concentrations (12.5%, 25%, 50%, 100%) of Gel10 or Gel10-Th200 extracts, while untreated cells served as negative controls. Following 24 h exposure under identical culture conditions, 10 µL CCK8 reagent was dispensed into each well. Spectrophotometric measurements at 450 nm wavelength were conducted using a microplate reader (SPARK 10M, Tecan, Switzerland).

For complementary viability assessment using dual fluorescence staining, L929 cells cultured in confocal dishes were treated with experimental extracts. After 24 h incubation, cellular staining was achieved through sequential addition of 4.5 µL propidium iodide (dead cell marker) and 10 µL calcein-AM (viable cell indicator). Dark-condition incubation at 37 °C was performed for 15min to facilitate dye activation. Cellular morphology and fluorescence patterns were ultimately visualized and documented using laser scanning microscopy (Axio Vert. A1, Carl Zeiss AG, Germany).

As the material acts directly on bone tissue, the CCK-8 method was used to supplement the test of osteocyte biocompatibility. Four concentrations of hydrogel extracts were prepared according to the above method. Then MC3T3-E1 and rBMSC cells were cultured in these extracts for 1, 3, and 5 days.

### 2.9 *In vivo* hemostasis and bone tissue repair tests

All protocols were performed in line with the rules and guidelines of the Association for Assessment and Accreditation of Laboratory Animal Care. The animal experiments were approved by Beijing Jishuitan Hospital (Approval No: 202308-02). The experimental animals were male domestic rabbits, each weighing 2.5 kg, purchased from Beijing Jinmuyang Experimental Animal Breeding Co., Ltd. The rabbits were randomly divided into three separate groups: control, Gel10, and Gel10-Th200. These animals were transferred to the housing facility at least 1 week prior to the operation to allow for their acclimatization.

In order to evaluate the hemostatic effect, a tibial defect model was established in rabbits. Before the surgical procedure, multiple sterile dry gauzes were weighed, and their mass was recorded as M_0_. The rabbits were anesthetized by intramuscular injection of ketamine and dexmedetomidine. After the rabbits were adequately anesthetized, the skin around the knee joint was prepared and disinfected. Subsequently, the skin and fascial tissues were incised layer by layer and the patella was dislocated. The proximal tibia was then exposed, and a 2.2-mm bone drill was used to create a bone defect penetrating the medullary cavity, resulting in medullary cavity bleeding. Hemostatic gel was immediately injected into the medullary cavity, while the control group received no material filling. The exuded blood was continuously wiped with pre-weighed dry gauzes. After the hydrogel injection, the bleeding situation of the bone defect was constantly observed to record the hemostasis time. The mass of the blood - absorbing gauzes was determined and designated as M_1_. The bleeding volume M was calculated according to the formula M = M_1_−M_0_. After the bleeding stopped, the area was continuously observed for an additional 15 min. Then, the patella was repositioned and the incisions were sutured. Three days later, the sutures were removed to check for any secondary bleeding in the body. At the end of the experiment, the rabbits were sacrificed.

For the osteogenesis tests, rabbits with tibial defects established for the experiments were randomly categorized into three groups. All the rabbits were weighed and then anesthetized. Similar to the tibial perforation model described previously, a tibial defect was created. The defect was filled with the prepared material in the experimental groups, whereas no material was placed in the control group. Subsequently, we sutured the incision and gave antibiotics within 3 days. Upon reaching a specific time point, rabbits in each group were respectively sacrificed, and the tibias were detached. After sampling at the second and fourth weeks respectively, the specimens were decalcified in EDTA for 4 weeks each and then embedded in paraffin. Subsequently, histological sections were prepared, tested with HE as well as Masson, and the resulting images were captured under a microscope. For the specimens collected at the fourth week, additional tests such as micro-CT scanning, blood routine analysis, and visceral organ safety evaluation were carried out.

### 2.10 Statistical analysis

Data were analyzed using GraphPad Prism software (version 8.4.3) and Origin (version 2022), with results expressed as the mean ± standard deviation (SD). Statistical significance among multiple groups was determined by two-way analysis of variance (ANOVA), where a *P*-value <0.05 was considered statistically significant. Significance levels are denoted as follows: ^*^ *P* < 0.05, ^**^ *P* < 0.01, ^#^ *P* < 0.001and ^&^ *P* < 0.0001.

## 3 Results and discussion

### 3.1 Preparation and characterization

**FIGURE 1:**
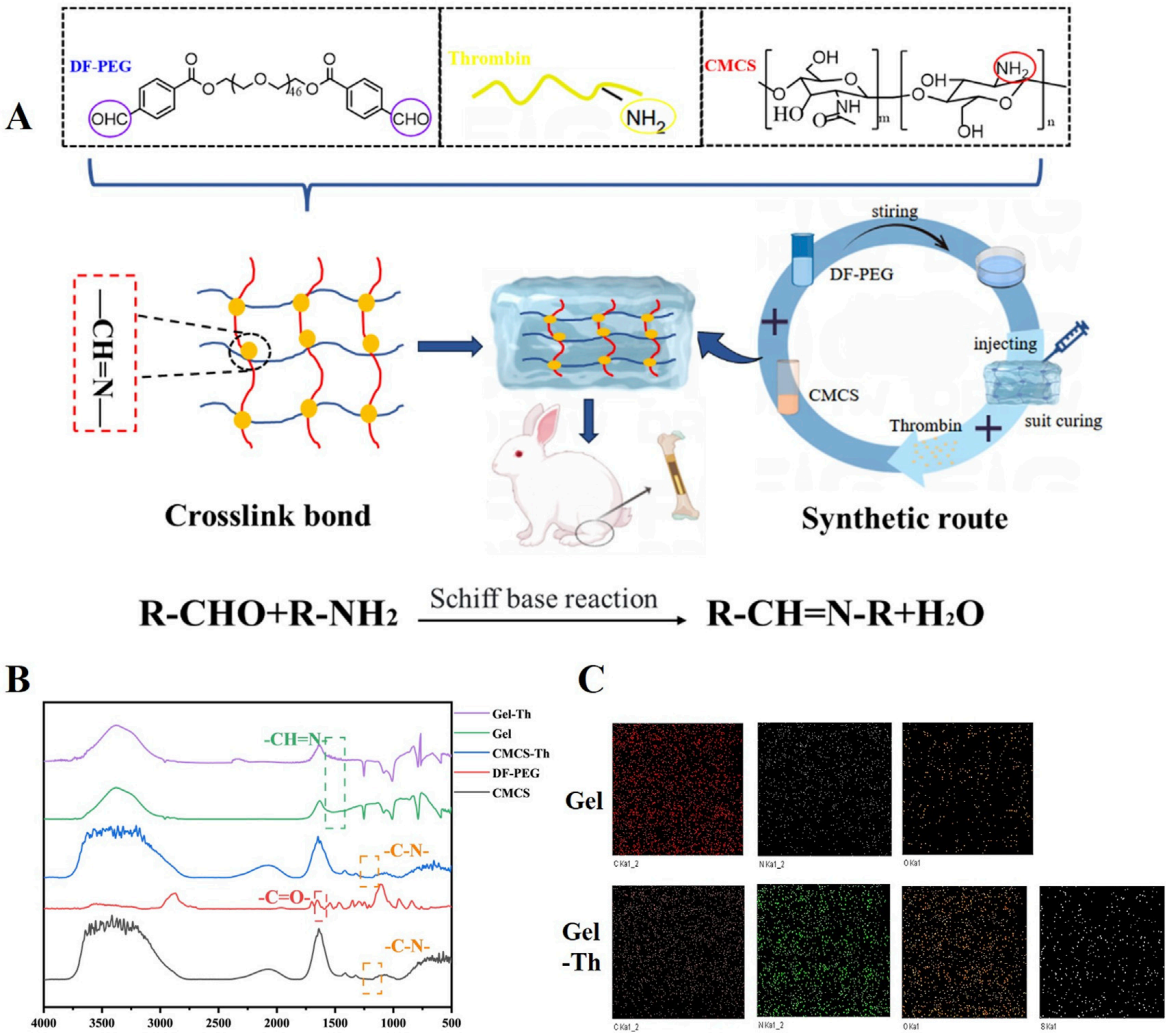
**(A)** Structural design and preparation of the hemostatic hydrogel. **(B)** FTIR spectroscopic profiles of DF-PEG, CMCS, CMCS-Th, Gel hydrogel, and Gel-Th hydrogel. **(C)** EDS of Gel hydrogel and Gel-Th hydrogel.

The Gel-Th hydrogel was fabricated through a Schiff base reaction (Figure 1A). The structural framework of the material was predominantly attributable to the covalent linkage between the aldehyde groups of DF-PEG and the amine groups of CMCS, which ultimately led to the formation of imine bonds within the molecular architecture. Additionally, thrombin contains amine functional groups, which contributed to a modest increase in the crosslinking strength of the hydrogel. The preparation process of the Gel-Th hydrogel can be delineated into two distinct steps: the first step involved selecting the optimal ratio of CMCS and DF-PEG for the hydrogel formulation, while the second step entailed the incorporation of thrombin into the selected DF-PEG/CMCS hydrogel (Gel hydrogel). The FTIR spectroscopic examination of the hydrogel manifested a distinctive absorption peak associated with the imine bond (-CH = N-) at a specific wavenumber of 1,550 cm^−1^ (Figure 1B; Supplementary Figure S1). This finding indicated the successful manufacture of the Gel and Gel-Th. Subsequent to the introduction of thrombin, the characteristic absorption trait of the imine bond was altered (as illustrated in Figure 1B), which suggested an elevation in the cross-linking compactness of the Gel hydrogel. The EDS characterization results (Figure 1C) indicated that the surface of the Gel hydrogel mainly contained three elements: C, N, and O. After adding thrombin, an additional element S appeared, which was due to the presence of disulfide bonds in thrombin. Moreover, all elements in both types of hydrogels were evenly distributed, suggesting that the materials had been uniformly mixed.

### 3.2 Physical property tests and selection of gel hydrogel

#### 3.2.1 Adhesion

**FIGURE 2:**
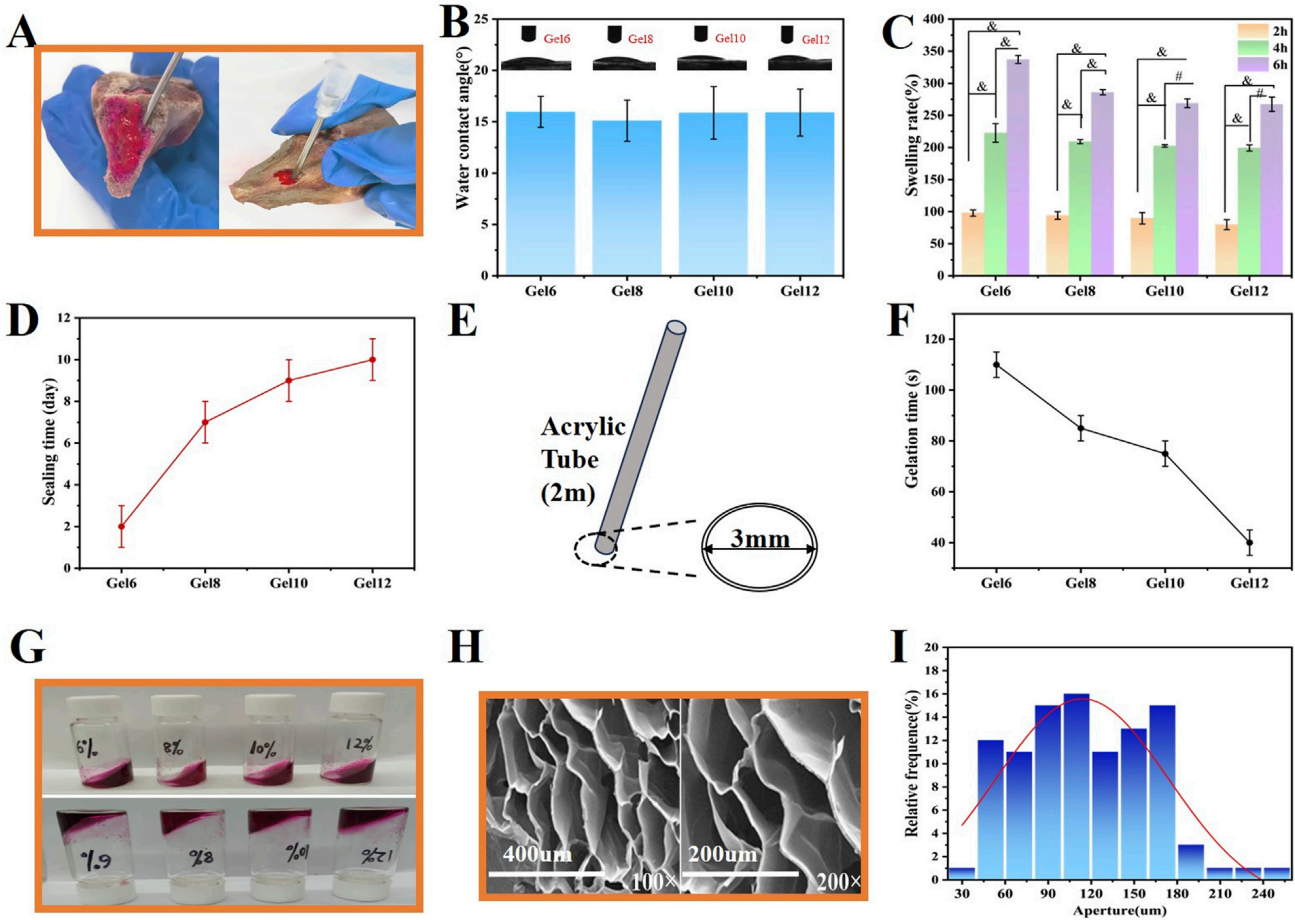
Physical property tests of Gel samples. **(A)** Qualitative characterization of the adhesive force, **(B)** Contact angle measurement, **(C)** Swelling rate of the Gel at 2, 4, and 6 h, **(D)** Liquid sealing time, **(E)** The models of liquid seal experiments, **(F)** Gelation time of DF-PEG and CMCS with varying concentrations in a room temperature, **(G)** The gelation time was determined using the small bottle tilting method (the upper and lower diagrams respectively showed the transformation from sol to gel), **(H)** SEM images of the Gel10 at magnifications of 100 × and 200 × , **(I)** Pore size analysis of freeze-dried Gel10. (*n* ≥ 3; ^*#*^ *P* < 0.001; ^*&*^ *P* < 0.0001)

Tissue adhesion implies that the hemostatic hydrogel can effectively seal the site of bleeding and resist being washed away by blood (Le Thi et al., 2017; Shen et al., 2024). The hydrogel remained firmly attached to the bone samples even after continuous water flushing (Figure 2A), indicating good adhesion between the hydrogel and the bone tissue. In addition, the adhesion effect was quantitatively analyzed by tensile test, and the results showed that Gel10 was the optimal ratio, and its degree of cross-linking balanced mechanical strength and structural toughness. For Gel6 and Gel8, due to insufficient cross-linking, they were difficult to withstand external forces, had poor structural stability, and were prone to losing their original shape due to deformation. As for Gel12, the degree of cross-linking was too high, the toughness of the material decreases, and stress concentration was easy to occur when stressed, leading to rapid structural failure (Supplementary Figure S2).

#### 3.2.2 Hydrophilicity

Hydrophilicity is the core link for dialogue between biomaterials and the biological environment, and its role runs through the entire process from molecular recognition to tissue integration, and in many hydrogel studies (Zhu et al., 2024; Li et al., 2025), static contact angles are widely accepted as a more reliable and reproducible indicator for characterizing initial hydrophilicity. The Gel samples exhibited a contact angle of approximately 15° (Figure 2B), indicating that all Gel samples possessed favorable hydrophilicity.

#### 3.2.3 Swelling

Effective swelling capacity can facilitate the absorption of blood, which is favorable for the aggregation of platelets and thereby triggers blood coagulation (Lee et al., 2023; Chen et al., 2025). The swelling characteristics of the Gel specimens were quantitatively evaluated *via* the determination of the percentage augmentation in mass across distinct temporal intervals (specifically, 2 h, 4 h, and 6 h). The swelling rate for each group increased over time. Concurrently, it was observed that the degree of crosslinking exhibited an inverse relationship with the swelling rate. At 6h, the swelling rate of Gel6 reached 337%, while that of Gel12 was 267% (Figure 2C).

#### 3.2.4 Liquid sealing

Medullary hemostasis primarily relies on the sealing capacity of the hydrogel following its curing process. Consequently, the assessment of liquid sealing is a crucial indicator of performance. The water-occluding efficacy of the Gel specimens was comprehensively appraised through the employment of an acrylic tube-based experimental model (Figure 2E) (Brückner et al., 2016). The relationship between the duration of liquid sealing and the degree of crosslinking of the hydrogel indicated that higher crosslinking resulted in extended sealing times. With the exception of the formulation with a 15:6 ratio, all other hydrogels demonstrated the ability to maintain sealing for more than 7 days (Figure 2D), thereby highlighting the effective sealing capability of the Gel samples.

#### 3.2.5 Gelation time

An ideal injectable hydrogel should possess an optimal gelation time for clinical applications (Xue et al., 2022; Zhai et al., 2024). When the gelation duration is excessively brief, the hydrogel is likely to experience the curing process inside the syringe, a phenomenon that has the potential to severely hamper its injectability. Conversely, if the gelation time is too slow, timely hemostasis may not be achieved. Therefore, we investigated the gelation time of the hydrogel at varying concentrations of CMCS. The gelation time of the Gel samples exhibited a decreasing trend with an increase in the concentration of the raw materials. With 15% (w/v) DF-PEG and CMCS concentrations ranging from 6% to 12% (w/v), the gelation times varied from 35 s to 115 s. Specifically, when mixing 15% (w/v) DF-PEG with 12% (w/v) CMCS at a 1:1 ratio, gelation occurred within a range of 35–45 s (Figures 2F,G). Additionally, the introduction of thrombin subsequently reduced the gelation time to some extent. Such a fast-curing speed cannot fully meet the requirements of the hemostatic operation time, which typically demands approximately 60 s. It may lead to problems such as the inability to completely cover the wound and the clogging of the syringe. In contrast, the remaining three concentrations satisfied the necessary operational time. Notably, a CMCS concentration of 10% yielded the fastest hemostatic performance compared to the other two concentrations.

Considering the preceding property evaluations, the concentrations of 15% (w/v) DF-PEG and 10% (w/v) CMCS were chosen as the most suitable ratio for the ensuing experiments.

#### 3.2.6 Micromorphology and pore size analysis

The mutually interconnected and intricately interpenetrating pores inherent in the Gel specimens endow them with the capacity to act as a physical barrier. Simultaneously, they promote the permeation of nutrients and the propagation of cells in a concurrent manner. Micrographs depicting the transverse cross-sections of the Gel and Gel-Th were presented in Figure 2H and Supplementary Figure S3, offering a detailed visualization of the internal structural features of the hydrogel. The results clearly demonstrated that the hydrogel possessed an exceedingly porous three-dimensional reticulated framework, and the aperture was all larger than 100 μm. Notably, the Gel10 featured large pores, with the majority of the pore sizes ranging from 90 μm to 180 µm (Figure 2I). Furthermore, we anticipate that these large pores will facilitate tissue ingrowth, thereby enhancing osseointegration during the bone regeneration process and stabilizing the mechanical integrity of the hydrogel (Mokhtarzadegan et al., 2021). The BET-BJH method will be employed in the future to supplement the analysis of the pore size and surface area of hydrogels, thereby enabling a more three-dimensional and multi-faceted demonstration of the porous structure of hydrogels.

#### 3.2.7 Thermal stability analysis

Analysis of the TGA-DTG data showed that neither Gel nor Gel-Th exhibited significant mass change in the initial stage (from room temperature to 100 °C), which is mainly attributed to the loss of moisture. In the temperature range of 200 °C–300 °C, the mass change primarily resulted from the decomposition of DF-PEG due to the breakage of its polymer chains. Within 300 °C–400 °C, the mass change was mainly caused by the decomposition of CMCS as a result of the breakage of its polymer chains. These results indicated that the hydrogel material decomposes only at high temperatures, and its structure remained intact under the mild *in vivo* environment. For the DSC analysis, we focused on detecting the melting temperature of the hydrogel, which was a critical temperature parameter for the prepared hydrogel. The melting temperature can be used to determine whether the material will melt *in vivo*, thereby affecting its sealing and hemostatic functions. The results showed that the material starts to melt at around 100 °C, which was much higher than the *in vivo* temperature, thus meeting our application requirements. The above two sets of test results demonstrated that the prepared hydrogel had good thermal stability for our intended use (Supplementary Figure S4).

### 3.3 Rheological measurement

The Gel10 screened was used as the main body to load thrombin, and the prepared Gel-Th was subjected to rheological property testing.

**FIGURE 3:**
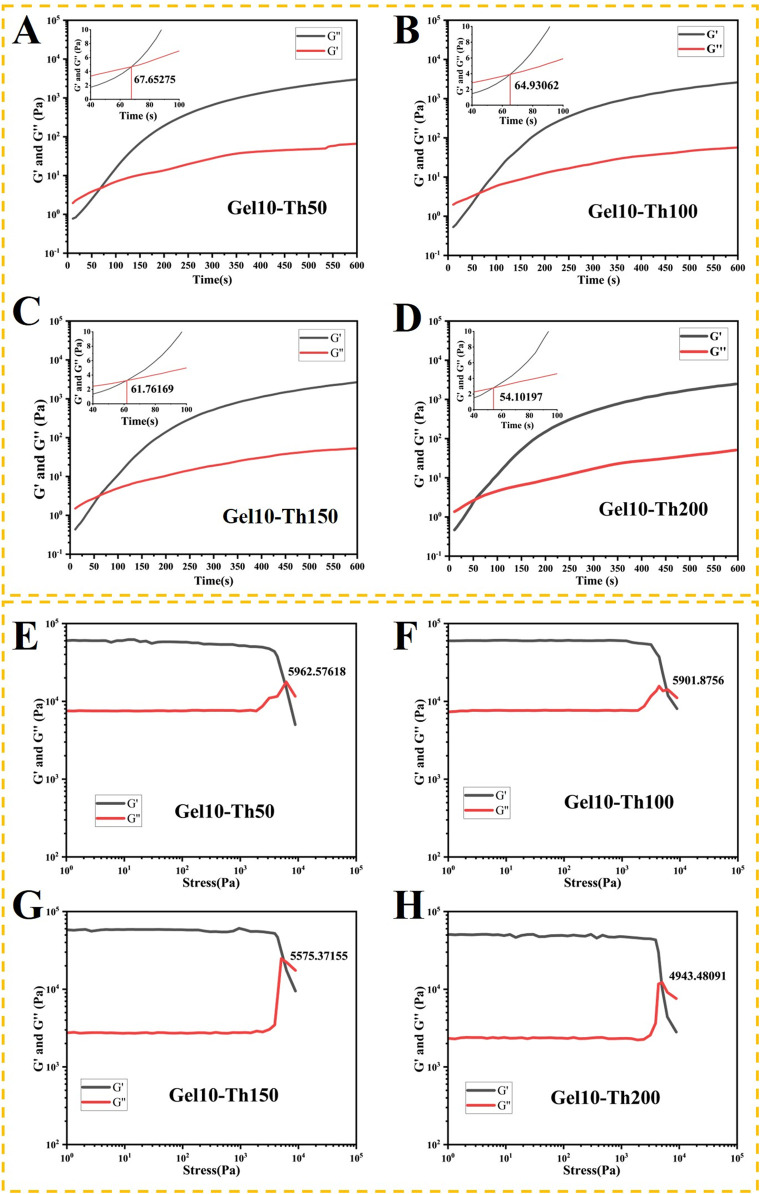
**(A–D)** The rheological properties of the hemostatic hydrogel were examined under a constant strain and frequency. The gelation point was determined as the crossover time point of the $G^{\prime }$ and ${G\;}^{\prime \prime }$ curves, **(E–H)** The rheological properties of the hemostatic hydrogel were measured at a constant frequency. The solation point was identified as the crossover stress point of the $G^{\prime }$ and ${\;G\;}^{\prime \prime }$ curves.

The $G^{\prime }$ and $G^{\prime \prime }$ of the Gel-Th hydrogel were determined as a function of time by rheometric methods. After the mixing of the raw materials, both the storage modulus ( $G^{\prime }$ ) and the loss modulus ( $G^{\prime \prime }$ ) manifested an ascending trajectory. Attributable to the formation of Schiff bases, the rate of increment of $G^{\prime }$ was more pronounced than that of $G^{\prime \prime }$ (M. Wang et al., 2023). The increase in the thrombin concentration led to a decrease in the gelation time and an improvement in both the storage and loss moduli of the Gel-Th specimens concurrently (Figures 3A–D). The instant at which $G^{\prime }$ superseded $G^{\prime \prime }$ was earmarked as the gelation point, which was congruent with the findings of the vial tilting test (Supplementary Figure S5A). When 15% (w/v) DF-PEG and 10% (w/v) CMCS solutions were blended at a 1:1 ratio, the gelation point was ascertained to be 73.0603 s. It is worth noting that the gelation time of Gel10-Th200 was 54.10197 s, approximately 19 s less than that of Gel10. Furthermore, the sharp increase in $G^{\prime }$ signified an augmentation of the hydrogel’s mechanical strength.

During the intramedullary nail implantation surgery, the hydrogel will flow out when it is squeezed. This is because the dynamic bonds will be broken when they are subjected to stress. The changing trends of the hydrogels in each group under stress were as follows: at small amplitude, $G^{\prime }$ and $G^{\prime \prime }$ remained basically unchanged, and $G^{\prime }$ was greater than $G^{\prime \prime }$ , indicating a stable cross-linked network state, this range was also known as the linear viscoelastic region. When $G^{\prime }$ intersected with $G^{\prime \prime }$ , the critical state point was reached, which marked the beginning of the destruction of the gel state. At large amplitude, $G^{\prime }$ became smaller than $G^{\prime \prime }$ , and the gel changed from an elastic-dominated “solid state” to a viscous-dominated “flow state”. This interval is called the non-inear viscoelastic range. It can be concluded that the hydrogel material we prepared exhibits elasticity under low stress and viscosity under high stress (Figures 3E–H; Supplementary Figure S5B). However, due to the different cross-linking degrees of each group, there were also differences in the stress critical points. The results showed that the stress critical point of Gel10 (6,336 Pa) was greater than that of the Gel-Th groups (5,963, 5,902, 5,575, and 4,943 Pa in sequence). This was because with the addition of thrombin, although the cross-linking degree was enhanced, the gel became hard and brittle, making it more likely to be damaged. In future work, we will pay attention to the impact of extrusion rate and carry out experiments on shear rate in combination with practical needs, we will also refine our research on the effects of amplitude oscillatory shear on hydrogels and supplement it with strain shear tests.

### 3.4 *In vitro* hemostatic evaluation and hemostasis mechanism

#### 3.4.1 *In vitro* hemostatic evaluation

**FIGURE 4:**
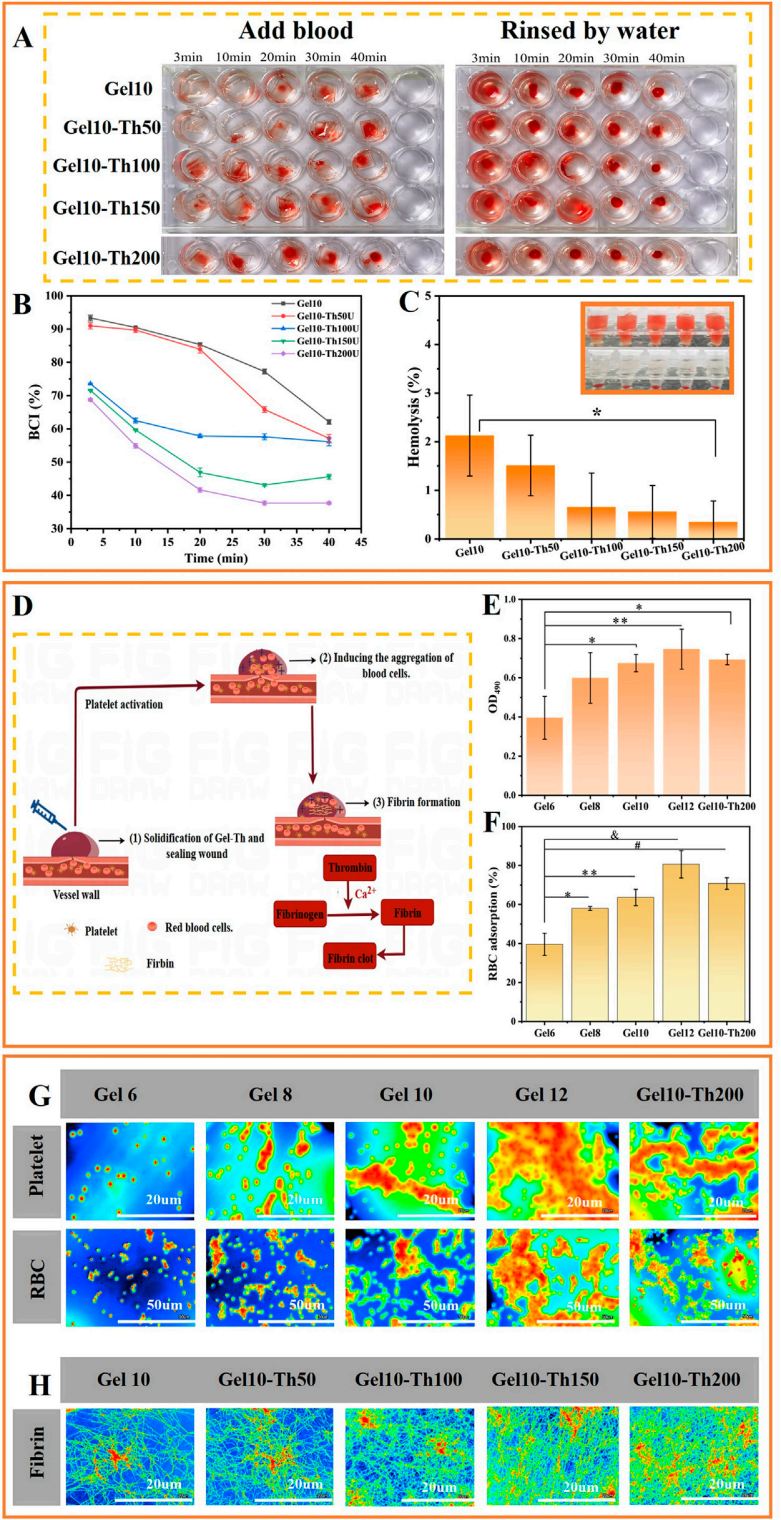
*In vitro* hemostatic evaluation and characterization of the hemostatic mechanism. **(A)** Images of the coagulation process, **(B)** BCI values, **(C)** Hemolysis rates, **(D)** The hemostatic mechanism processes, **(E,F)** Quantitative analysis of the adhesion of Platelets and red blood cells, **(G)** Qualitative analysis of the adhesion of blood cells, **(H)** The study of fibrin. (*n* ≥ 3; *P < 0.05; **P < 0.01; ^*#*^ *P* < 0.001; ^*&*^ *P* < 0.0001).

To evaluate the hemostatic attributes of Gel-Th specimens with precision, *in vitro* coagulation assays were executed using the blood clotting index (BCI) test. At the onset, a qualitative evaluation of the materials was conducted by subjecting them to incubation with blood and subsequently performing a rinse with deionized water. A lighter coloration of the water was indicative of more rapid clotting (Shi et al., 2020). The water encircling the Gel10 assumed a red coloration on account of the presence of non-coagulated blood, suggesting a limited blood coagulation competence of this material. In accordance with an increase in thrombin content, the gradual fading of the color around the Gel-Th specimens signified that the thrombin-laden hydrogel substantially reduced the blood clotting duration (Figure 4A). Furthermore, a quantitative analysis of the BCI was carried out, wherein a higher index was indicative of a diminished capacity for blood coagulation (Yu et al., 2023). The results of the investigation demonstrated that the BCI of the Gel-Th specimens decreased in tandem with an increasing thrombin concentration (Figure 4B). Notably, the BCI for the Gel10-Th200 (37.72%) was significantly lower than that of the Gel10 (62.08%) at the final measured time, suggesting the Gel10-Th200 enhanced the coagulation effect by promoting the conversion of fibrinogen.

Hemocompatibility represents a pivotal parameter in the formulation of efficacious hemostatic agents; thus, the hemolysis ratio was evaluated (Wang et al., 2017). As the concentration of thrombin increased, the hemolysis rate gradually decreased (Figure 4C). The hemolysis ratio for the Gel10-Th200 was approximately 0.34%, whereas the ratio for the Gel10 was higher (approximately 2.13%). These findings indicated that the Gel10-Th200 demonstrated good hemocompatibility, in alignment with national and international standards, which classify hemolysis ratios below 5% as indicative of excellent hemocompatibility.

#### 3.4.2 *In vitro* hemostasis mechanism

The hemostatic mechanism encompasses the intricate interactions among erythrocytes, thrombocytes, coagulation factors, and other associated substances (Nepal et al., 2023). The hemostatic mechanism of CMCS/DF-PEG/thrombin hydrogel can be categorized into three primary processes (Figure 4D). Firstly, once the hydrogel solidified, its physical properties effectively occluded the wound, facilitating mechanical hemostasis. Secondly, the positively - charged surface of the hydrogel established an electrostatic “gravitational field” that prompted the aggregation of blood cells. Ultimately, through the synergistic effect of thrombin, fibrinogen was swiftly transformed into a three-dimensional fibrin reticulum, which further entrapped and encapsulated blood corpuscles, culminating in the formation of a stable blood clot. We devised the subsequent experiments to conduct a more in-depth exploration of the hydrogel’s function in the processes of primary and secondary hemostasis.

It is widely acknowledged that the aggregation of blood corpuscles assumes a pivotal role in primary hemostasis (Fan et al., 2023). The absorbance at 490 nm was measured, with higher absorbance values (indicating deeper coloration) representing greater LDH concentrations and a higher number of platelets adsorbed on the sample surface. This assay facilitated the quantitative assessment of the thrombocyte adhesion capacity on the specimen surface. The findings demonstrated that with an increase in the concentration of CMCS (Figure 4E), which led to a greater quantity of cations, the absorbance values determined for Gel8, Gel10, and Gel12 were 0.59915, 0.6752, and 0.74591 respectively. This indicated that these gels exhibited a better platelet aggregation capacity than Gel6 (0.39577). Moreover, due to the stimulation of blood cell aggregation by thrombin, the absorbance value of Gel10-Th200 (0.69312) showed a slight increase compared to Gel10 (0.6752).

The absorbance of the hydrogel group and the control group at 540 nm was compared, and the adhesion effect of erythrocytes was quantitatively analyzed. From the results (Figure 4F), Gel6, Gel8, Gel10, and Gel12 increased sequentially, being 39.6, 57.9, 63.6 and 80.6 respectively. Gel10-Th200 was also between Gel10 and Gel12, which explained the promoting effect of chitosan and thrombin components on erythrocyte adhesion.

Concurrently, the aggregation of blood cells on the material’s surface was visualized *via* laser-scanning confocal microscopy. The two-dimensional images were supplemented (Supplementary Figure S6A). There was a notable increase in the quantity of aggregated blood cells with the rising content of CMCS (Figure 4G). This phenomenon can be attributed to the positively charged amino groups in CMCS, which attracted negatively charged blood cells. It was worth mentioning that the hydrogel, cross-linked from 15% DF-PEG and 10% CMCS, exhibited a slight enhancement in the adsorption of blood cells when loaded with thrombin. This effect occurred because thrombin stimulated the coagulation process, thereby promoting the aggregation of blood cells. The phenomenon was consistent with the quantitative analysis results.

A highly dense fibrin reticulum, which effectively entrapped and securely encapsulated blood cells, gradually emerged on the surface of Gel-Th (Figure 4H). Conversely, only a tenuous fibrin meshwork was discernible on the surface of Gel. This marked disparity could potentially be attributed to the fact that thrombin enhanced the interfacial stimulatory capacity of Gel-Th, thereby potentially catalyzing the conversion of fibrinogen into fibrin, a process that is well-documented in reference (Wolberg and Campbell, 2008). This phenomenon signified the commencement of secondary coagulation. These empirical findings substantiated that thrombin within Gel-Th exerted a significant and notable influence on blood coagulation at the wound-material interface. The two-dimensional images were supplemented (Supplementary Figure S6B).

### 3.5 Drug release and *in vitro* degradation

**FIGURE 5:**
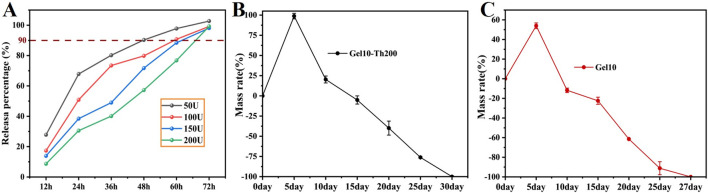
Drug release and *in vitro* degradation. **(A)** Drug release of Gel-Th testing every 12 h, **(B,C)** *In vitro* degradation of Gel10 and Gel10-Th200.

At the beginning, the contact of blood with thrombin immediately initiated the occurrence of blood coagulation. Subsequently, the thrombin loaded in the Gel-Th would be released slowly. The continuous release can maintain the stability of the hemostatic effect. The *in vitro* simulation of the release of Th in Gel-Th was conducted, and the results were shown (Figure 5A). Th50 and Th100 experienced burst release within the first 36 h and were almost completely released by 72 h Th200 and Th150, on the other hand, were gradually released and were also almost fully released by 72 h. This might be because Th200 and Th150 hydrogels contained more thrombin, resulting in a relatively higher degree of cross-linking in the system, slower water absorption and swelling, and thus a slower release of thrombin from the hydrogel. The drug release mechanism was further determined by the kinetic model, and the results were showed (Supplementary Table S1), Gel10-Th50 conformed to the first-order kinetic model, Gel10-Th100 conformed to the Ritger-Peppas model, and both Gel10-Th150 and Gel10-Th200 conformed to the zero-order kinetic model (the core characteristics and formulas of each kinetic model were included in the Supplementary Material). The verification results of the kinetic models can further determine the release mechanism of each drug-loaded hydrogel, which were also consistent with the cumulative release curves. While the hemostatic efficacy indirectly confirms the activation and functionality of the released thrombin, future work involving direct quantification of thrombin activity and its release kinetics will be essential to fully elucidate the detailed pharmacodynamic profile for clinical translation.

After the above tests, Gel10-Th200 was selected as the optimal formula for subsequent performance verification. The *in vitro* degradation behavior of the Gel10 and Gel10-Th200 hydrogels was meticulously appraised *via* the gravimetric method in a simulated physiological environment (Figures 5B,C). During the incipient phase of the degradation process, there was an increase in both the volumetric and mass dimensions of the hydrogels. This observable phenomenon could be attributed to the degradation property of imine-based hydrogels that is triggered by swelling, a process that commonly occurs within liquid-filled surroundings. As time passed, the hydrogels underwent a progressive degradation, and the mass of the specimens exhibited a continuous decrease. By the 20th day, the hydrogels had undergone a degradation of roughly 50%, and they were on the verge of being entirely degraded by 27 days. The addition of thrombin further enhanced the cross-linking degree, making the Gel10-Th200 hydrogel degrade more slowly. The fabricated hydrogels featuring appropriate biodegradability hold promise for potential applications. The healing cycle of bone defects is relatively slow, taking 2–3 months (Barba et al., 2019). Therefore, hemostatic materials should ideally degrade within this period to avoid interfering with bone healing. In the future, we will gradually conduct in-depth studies on simulated degradation and supplement our research in the degradation process of biomaterials, as such tests would complement our previous macroscopic tests.

### 3.6 *In vitro* and *in vivo* biosafety testing

#### 3.6.1 *In vitro* safety

**FIGURE 6:**
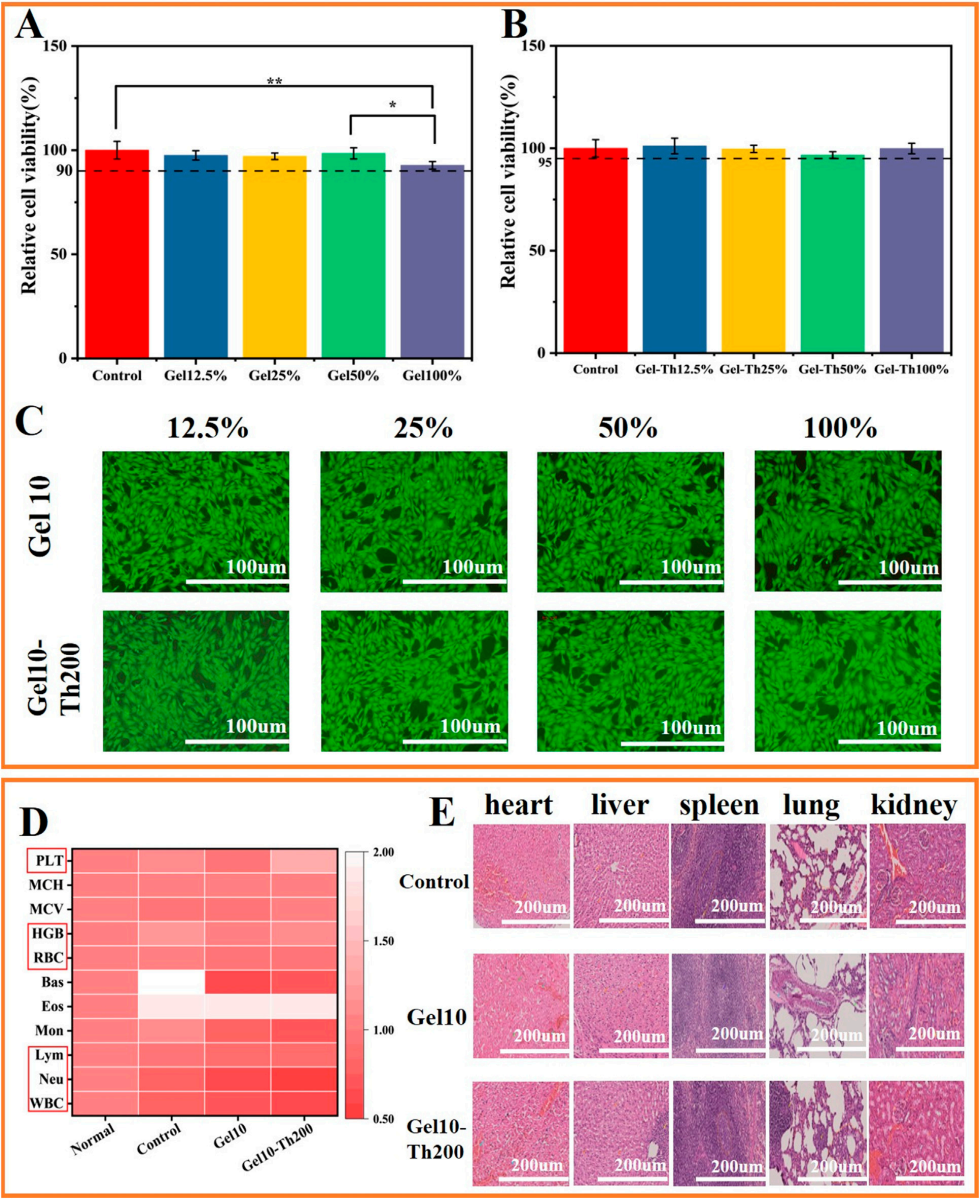
*In vitro* and *in vivo* biosafety testing. **(A,B)** Cell viability of Gel and Gel-Th, **(C)** Fluorescence micrographs of L929 cells treated for 24 h (Fluorescence images of living cells). **(D)** Blood routine tests, **(E)** HE tissue sections of internal organs (heart, liver, spleen, lung, kidney) were used to analyze the *in-vivo* safety of animals. (*n* ≥ 3; *P < 0.05; **P < 0.01).

Considering that Gel10 and Gel10-Th200 were designated for direct utilization on hemorrhagic wounds, a comprehensive evaluation of their biocompatibility was carried out. After a 24-h period of incubation, the viability of cells within all experimental groups surpassed 90%, and no discernible cytotoxicity was identified in any of the groups (Figures 6A,B). The outcomes of the qualitative cytotoxicity examination following 24 h of incubation were presented. The green fluorescence served as an indication of viable cells within Gel10 and Gel10-Th200, while only a negligible quantity of non-viable cells (characterized by red fluorescence) was present throughout all the experimental sets (Figure 6C; Supplementary Figure S7A). These empirical observations substantiated that all the experimental groups manifested commendable cytocompatibility. Since Gel-Th is used for hemostasis in bone tissues, the biocompatibility of the hydrogel with bone cells was tested. There was no significant difference between the hydrogel group and the control group (Supplementary Figure S7B), indicating that the hydrogel is non-toxic to bone cells.

#### 3.6.2 *In vivo* safety

The venous blood was tested using an automatic blood analyzer, and the blood routine indicators were mainly the following: WBC, Neu, Lym, RBC, HGB and PLT. The test results showed that the indicators of all groups were within the normal range, indicating that the rabbits did not have such as inflammation, anemia, abnormal coagulation and other phenomena (Figure 6D). In addition, the *in vivo* safety was further confirmed through HE staining of five internal organs for observation (Figure 6E), indicating that there was no obvious inflammatory condition in the internal organs of each group.

### 3.7 *In vivo* hemostasis and bone tissue repair evaluation

#### 3.7.1 *In vivo* hemostasis evaluation

**FIGURE 7:**
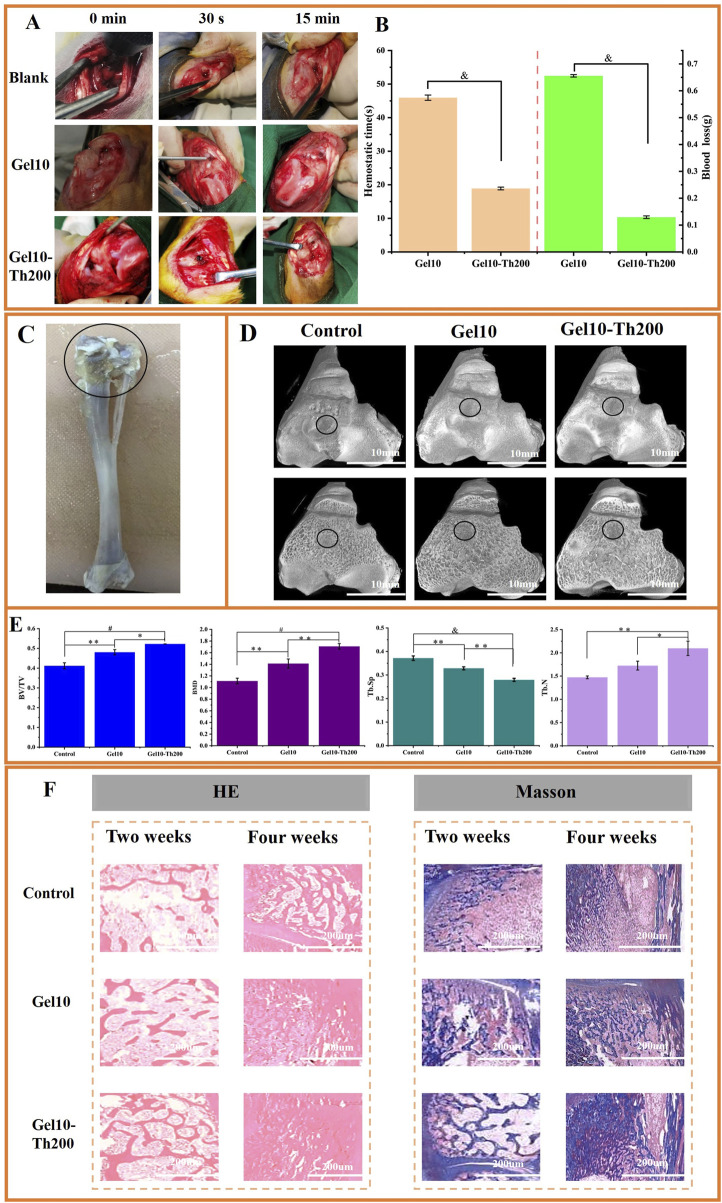
*In vivo* hemostasis and bone tissue repair evaluation. **(A)** The hemostasis process of tibial defect (Blank control group, Gel10 group, Gel10-Th200 group), **(B)** Hemostatic time and blood loss of Gel10 and Gel10-Th200, **(C)** The part of the tibia material (The circled part was the drilled end), **(D)** Micro-CT top view images of three groups at the implantation site, **(E)** Quantitative analysis of micro-CT data for BV/TV, BMD, Tb. Sp and Tb. N. All data were obtained 4 weeks after implantation, **(F)** Histopathological analysis (HE, Masson) was performed during the second and fourth weeks. (*n* ≥ 3; *P < 0.05; **P < 0.01; ^*#*^ *P* < 0.001; ^*&*^ *P* < 0.0001).

To conduct a more in-depth assessment of the impacts of Gel-Th on hemostasis and bone restoration, rabbit models of bone bleeding and bone defects were utilized. Gel10 and Gel10-Th200 were administered to bleed tibial defects to evaluate their hemostatic efficacy (Figure 7A; Supplementary Figure S8). It was obvious that Gel10 and Gel10-Th200 could quickly stop bleeding and there was no secondary bleeding within 15 min and 3 days after hemostasis. However, in the blank group, from the beginning of drilling, there was still slight blood seepage after 15 min of pressing with gauze. After suture, blood clotted by itself, and hemostasis was also achieved later. The blood clotting times in the Gel10 and Gel10-Th200 groups were 45.95333 ± 0.77976 s and 18.9 ± 0.40825 s. Moreover, the initial occlusion of the bleeding site using Gel10 and Gel10-Th200 led to blood loss of 0.65567 ± 0.00464 g and 0.12933 ± 0.00478 g respectively (Figure 7B). This disparity was attributable to the fact that Gel10-Th200 exhibited a substantial capacity for secondary hemostasis, whereas the hemostatic efficacy of Gel10 primarily relied on its physical sealing characteristic.

#### 3.7.2 *In vivo* bone tissue repair evaluation

The bone’s regenerative capacity is vital for successful implantation. Through Micro-CT scanning of the entrance site of the implant (Figure 7C). New bone tissue continued to grow around the hydrogel, and the bone repair effect of Gel10-Th200 was the best (Figure 7D). Secondly, in order to quantitatively analyze the ability of bone growth, the BV/TV, BMD, Tb. SP and Tb. N of this part of the bone tissue were tested (Figure 7E). It can be seen that the BV/TV, BMD and Tb. N (bone volume fraction, bone density, number of trabeculae) of the Gel10-Th200 group were all greater than those of Gel10 and the control group, but the Tb. SP (trabecular separation) was less than those of the other two groups, indicating that the Gel10-Th200 group had better bone repair ability. The good biocompatibility of the hydrogel itself and the repair-promoting ability of thrombin component can better promote wound repair.

Histological analysis was performed at the second and fourth weeks post-implantation of the hydrogel. The tibia was stained with HE and Masson to assess inflammation and osteogenic response *in vivo* (Figure 7F). As shown by HE staining, no obvious inflammatory response was observed in all groups, and Gel10-Th200 gradually formed abundant bone trabeculae and blood vessels. According to Masson staining, the area surrounding the Gel10-Th200 was characterized by abundant bone tissue and denser connective tissue. Studies have proven that thrombin can activate the receptors on cells and promote the proliferation and migration of fibroblasts and endothelial cells. This is conducive to the formation of collagen and blood vessels, providing favorable conditions for the repair of bone tissue (Moura et al., 2024; Kleeschulte et al., 2018). In conclusion, Micro-CT and histological staining analysis indicated that the Gel10-Th200 hydrogel had excellent safety and tissue repair capability. Future studies will involve a comprehensive comparison between Gel10-Th200 and currently available commercial products, as well as other advanced hemostatic hydrogels reported in the literature. We will also actively investigate the microenvironmental mechanisms underlying Gel10-Th200 roles in both hemorrhage control and osteogenic repair. More large-animal models—such as sheep, canine, or porcine models, which better approximate human bone anatomy and pathological conditions—will be employed to enable in-depth mechanistic validation and thorough performance evaluation. These steps are essential to establishing a solid foundation for the eventual clinical translation of our material. Furthermore, we aim to optimize the material’s properties to better synchronize with the tissue regeneration process, and explore the integration of multiple functions to develop a new generation of injectable, intracavity hemostatic hydrogels capable of *in situ* gelation. These next-generation systems are intended to be intelligent, self-adapting, and capable of multi-target interventions.

## 4 Conclusion

Injectable hemostatic materials that can fill narrow, elongated and complex wounds and achieve hemostasis without external compression are currently being developed through numerous efforts. In this research, we have presented a novel utilization of the Gel-Th hydrogel as a hemostatic agent. The Gel-Th hydrogel demonstrated injectability and enhanced mechanical characteristics after *in-situ* curing, which were crucial for wound coverage and hemostasis. Furthermore, *in vitro* studies indicated that Gel-Th hydrogel can induce secondary hemostasis. Based on the analysis of the dynamic model, thrombin was gradually released from the gel, which facilitated the conversion of fibrinogen to fibrin. The Gel-Th hydrogel demonstrated the effects of hemostasis and tissue repair in a rabbit marrow cavity hemorrhage model. In summary, we deduce that the Gel-Th hydrogel represents an efficacious hemostatic material featuring a straightforward preparation methodology. Although the above research content has met the current needs, we will continue to explore in the future. For example: given the hydrogel has the potential for injectability, and research on hemostatic materials of hydrogels for injection use will be carried out in the future, it is necessary to further improve their rheological test characterization to investigate the effects of shear rate and strain magnitude on the extrusion performance of hydrogels. Meanwhile, in-depth research on the microstructure of hydrogels is also required, which specifically includes exploring the changes in microstructure caused by degradation and analyzing the pore size and surface area of hydrogels using methods such as BET-BJH. Moreover, the optimized hydrogel materials will exhibit superior performance and can be further promoted as next-generation intramedullary hemostatic materials.

## Data Availability

The raw data supporting the conclusions of this article will be made available by the authors, without undue reservation.
