# Supplementary material for: Injectable in-situ curable hydrogel for medullary cavity hemostasis
Source: Front Bioeng Biotechnol. 2025 Sep 23;13:1658768. doi: 10.3389/fbioe.2025.1658768 (PMC12500688; doi:10.3389/fbioe.2025.1658768)
Supplement: Supplementary file 1 [file Supplementaryfile1.docx]

**Supplementary Information for**

**Injectable in-situ curable hydrogel for medullary cavity hemostasis**

Xiangxiao Meng ^1, +^, Fei Liu ^2, +^, Yichun Dou ^1^, Chao Shi ^3^, Jingshuang Zhang ^2^, Xieyuan Jiang ^2,^ * and Rui Shi ^2,^ *

^1^ State Key Laboratory of Organic-Inorganic Composites, College of Materials Science and Engineering, Beijing University of Chemical Technology, Beijing 100029, PR China

^2^ Beijing Jishuitan Hospital, Capital Medical University; Beijing Research Institute of Traumatology and Orthopaedics, Beijing, 100035, P.R. China

^3^ Research Center for Intelligent Design and Manufacturing of High-End Oil and Gas Equipment, College of Mechanical and Transportation Engineering, China University of Petroleum, Beijing 102249, PR China

* Authors to whom correspondence should be addressed.

E-mail address: [jxytrauma@163.com](mailto:jxytrauma@163.com) (Xieyuan Jiang), [shirui@jst-hosp.com.cn](mailto:shirui@jst-hosp.com.cn) (Rui Shi).

+ These authors contributed equally to this work and share first authorship.


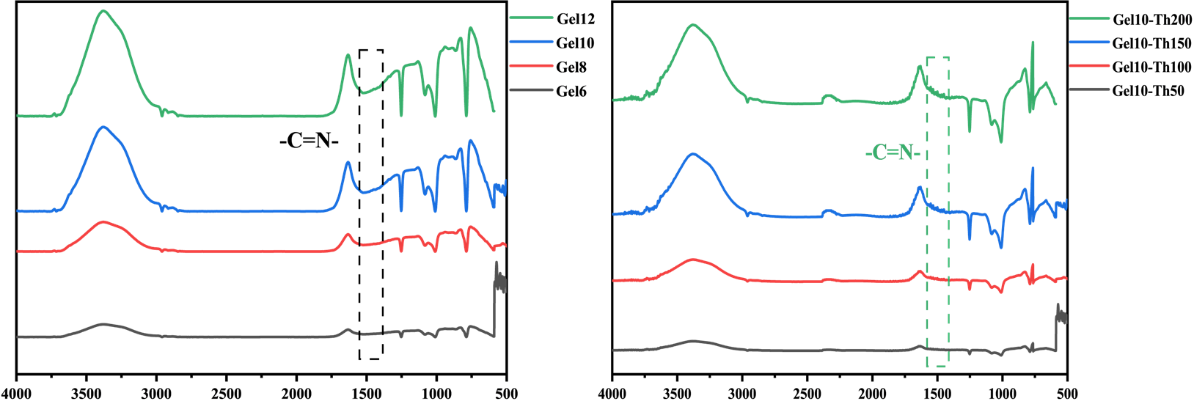


**Fig. S1.** Infrared spectra of each group of formulations


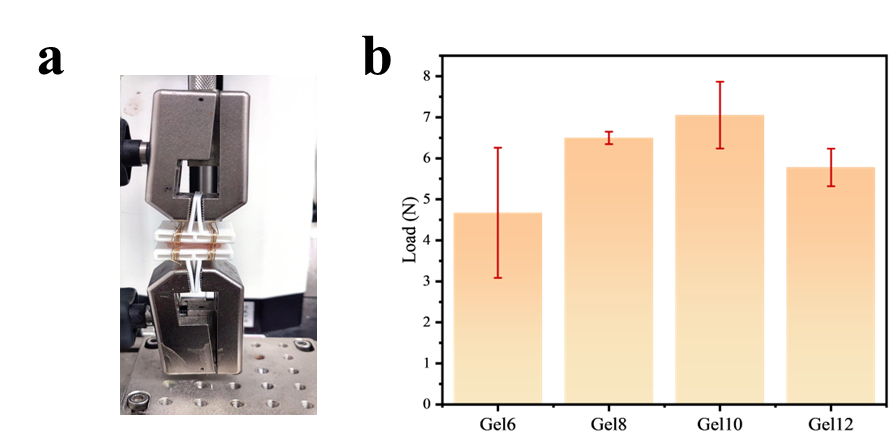


**Fig. S2.** Quantitative Analysis of Adhesion Effect. (a) Construction of stretching device. (b) Quantitative analysis of tensile stress.

**
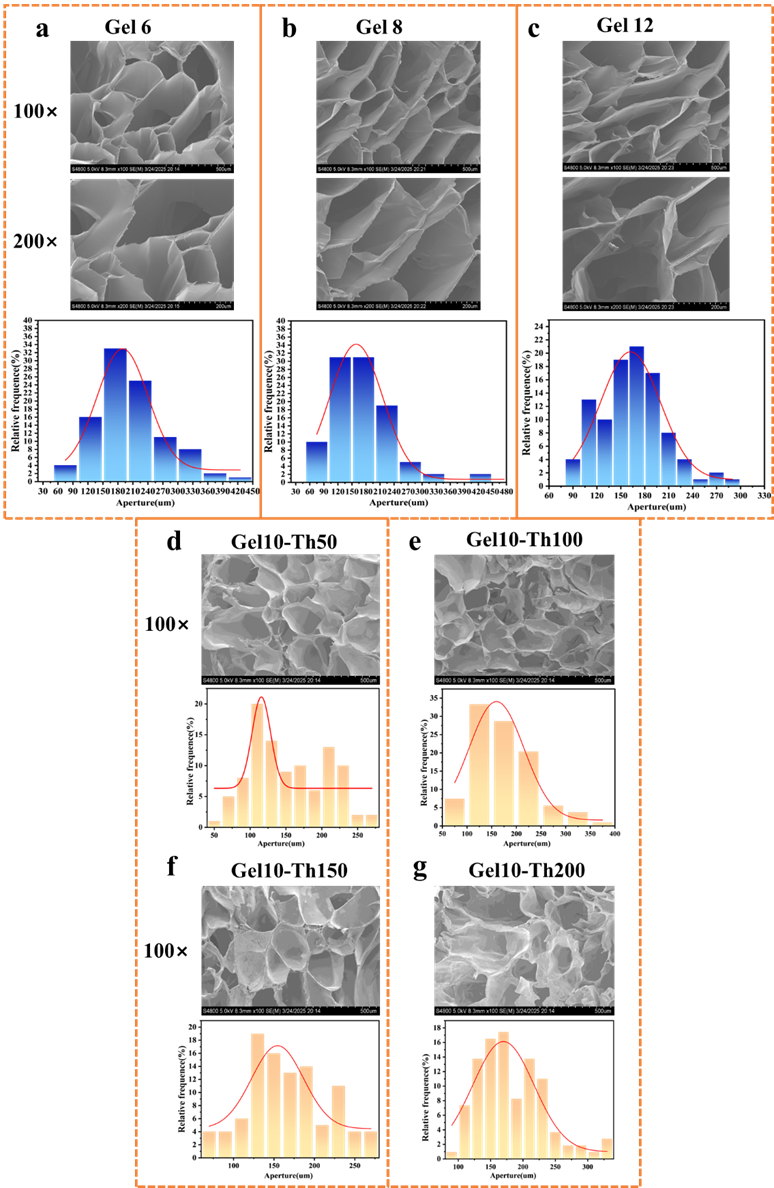
**

**Fig. S3.** （a-c）Surface morphology analysis of Gel samples. Gel6, Gel8 and Gel12 aperture morphology and pore size. （d-g）Surface morphology analysis of Gel-Th samples. Gel10-Th50, Gel10-Th100, Gel10-Th150 and Gel10-Th200 aperture morphology and pore size.


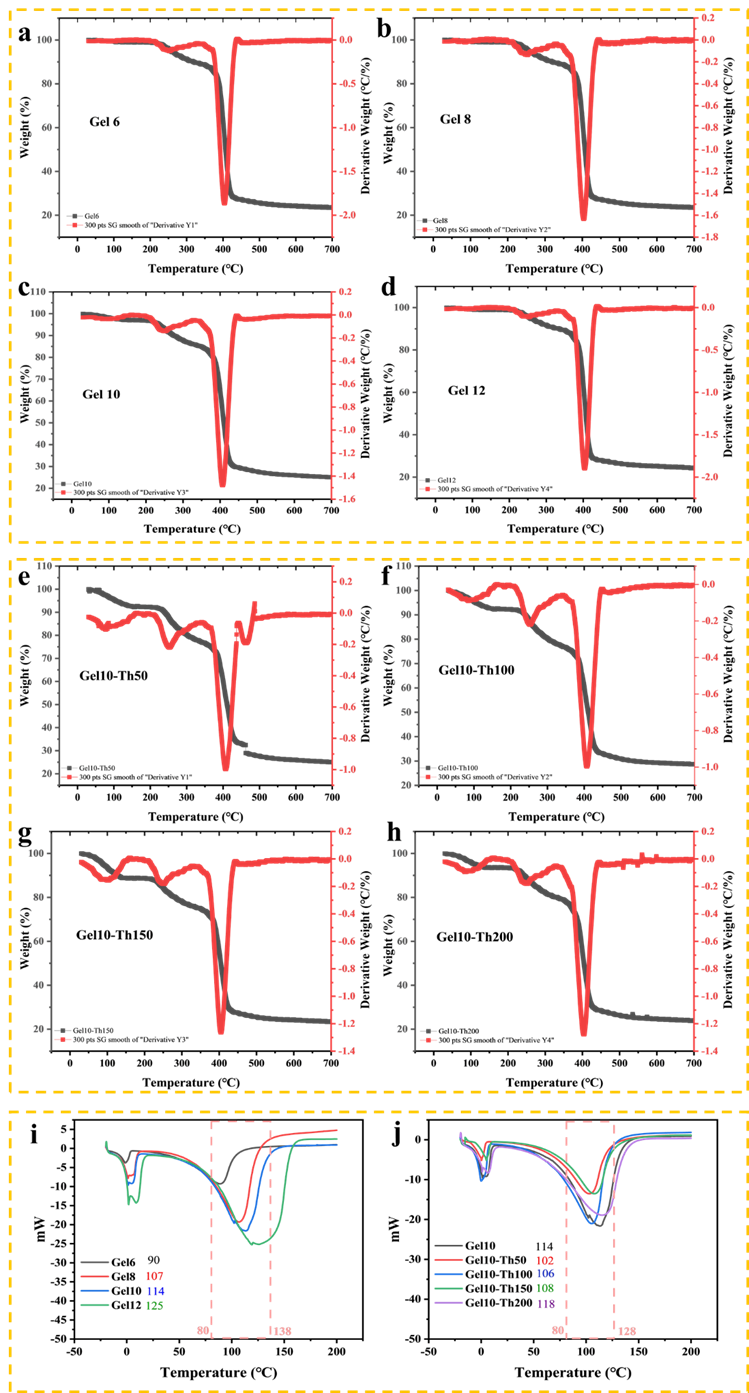


**Fig. S4.** (a-d) Thermogravimetric Analysis-Derivative Thermogravimetric Analysis of Gel. (e-h) Thermogravimetric Analysis-Derivative Thermogravimetric Analysis of Gel-Th. (i) Differential Scanning Calorimetry of Gel. (j) Differential Scanning Calorimetry of Gel-Th.


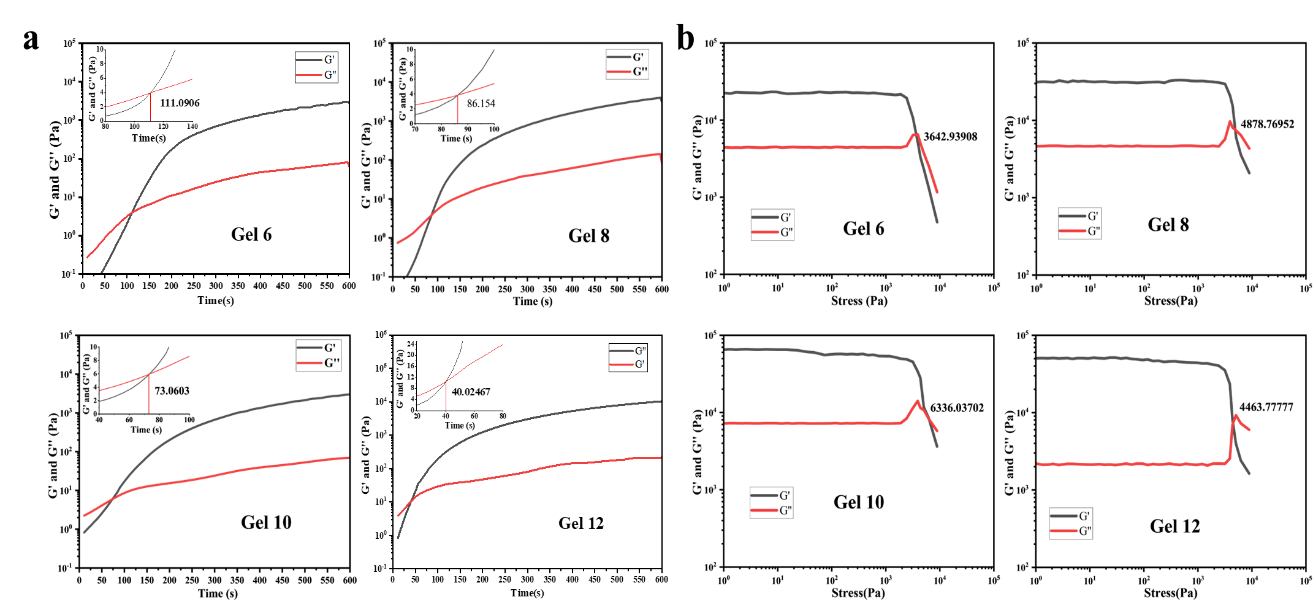


**Fig. S5.** (a) The rheological tests quantitatively analyze the gelling time of Gel, which is consistent with the test time of the vial tilt method. (b) The rheological tests quantitatively analyze the solation point of Gel.


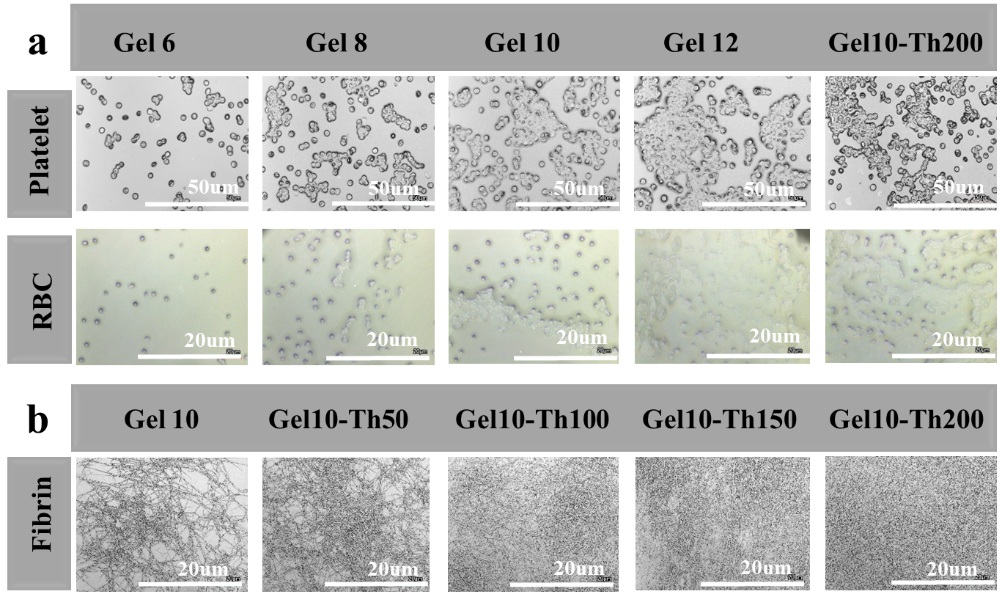


**Fig. S6.** Representation of two-dimensional images. (a) Qualitative analysis of the adhesion of blood cells for Gel and Gel-Th. (b) The study of fibrin for Gel and Gel-Th.


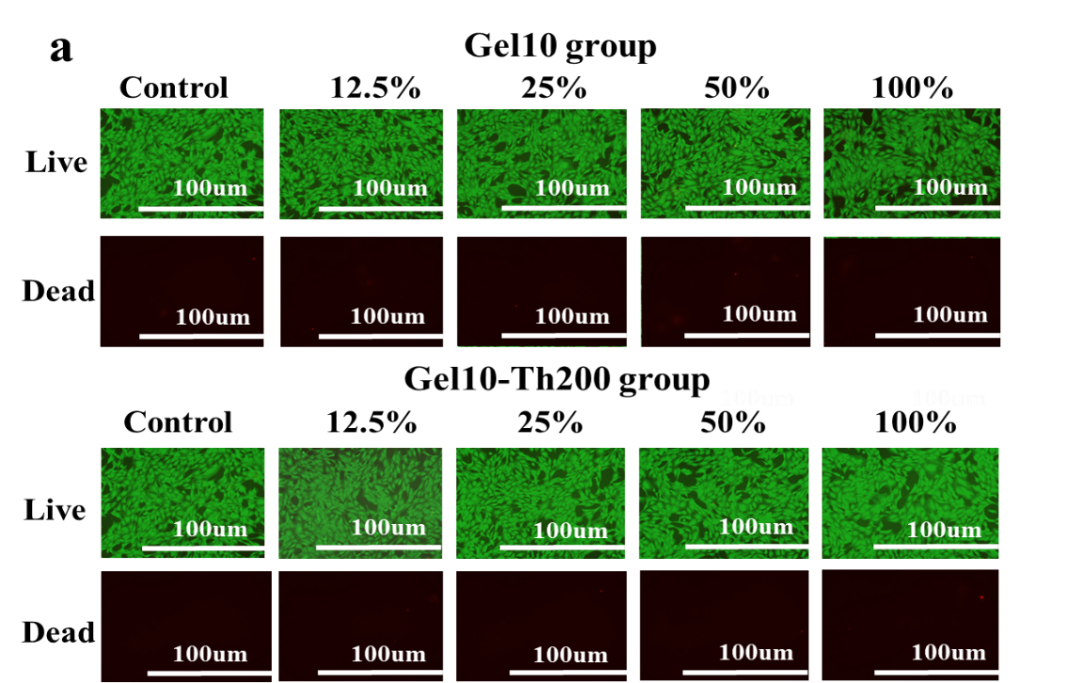


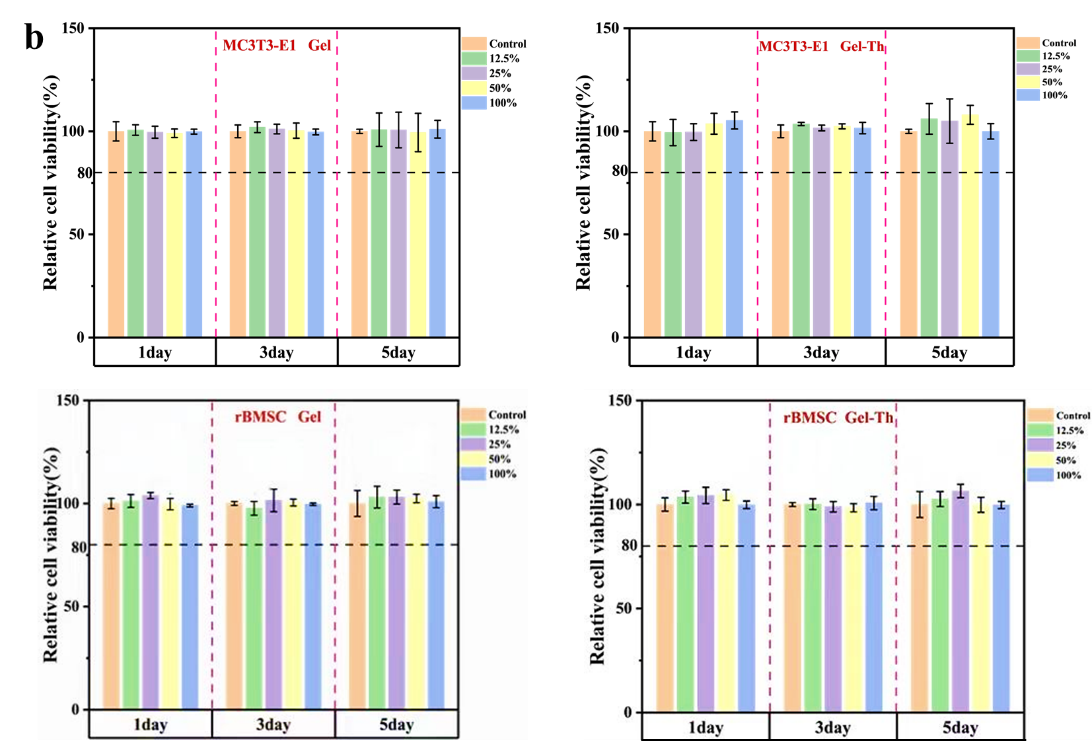


**Fig. S7.** (a) Fluorescence micrographs of L929 cells treated for 24 h, (b) Cell proliferation results of MC3T3-E1and rBMSC for 1 day, 3 days and 5 days (*n* ≥ 3).


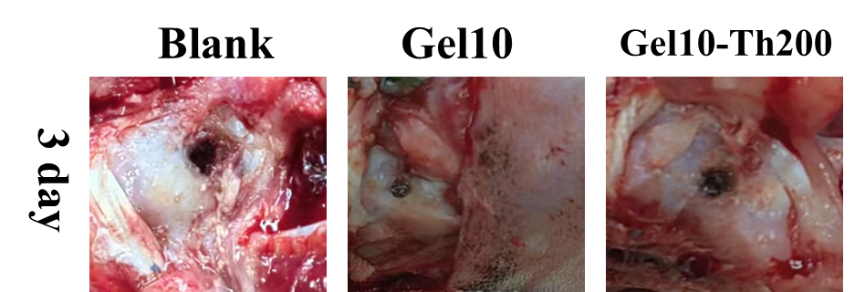


**Fig. S8.** After three days, open the package and observe the effect of hemostasis in the body.

| **Gel10-Th50** | |
| --- | --- |
| Zero-order kinetic model  R^2^=0.8859 | 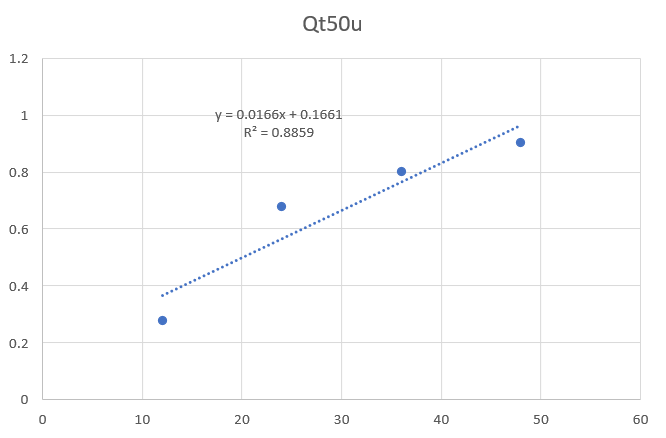 |
| Higuchi model  R^2^=0.9394 | 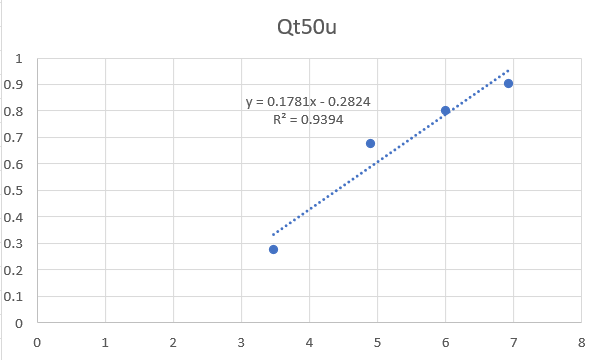 |
| First-order kinetic model  R^2^=0.9917 | 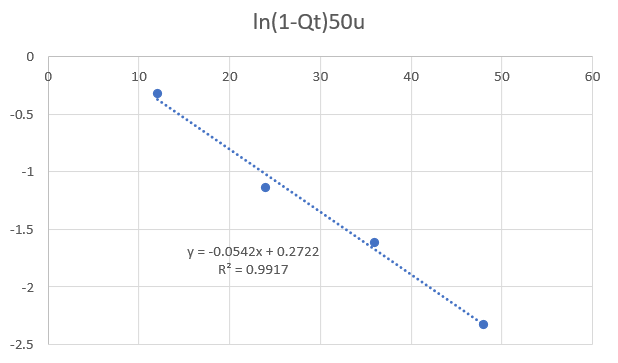 |
| Ritger-Peppas model  R^2^=0.928 | 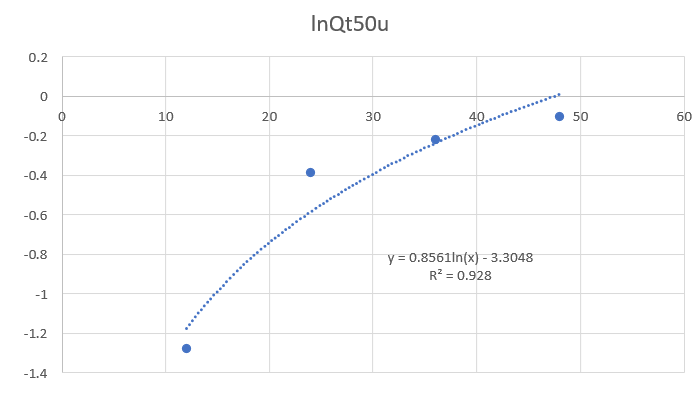 |

| **Gel10-Th100** | |
| --- | --- |
| Zero-order kinetic model  R^2^=0.9061 | 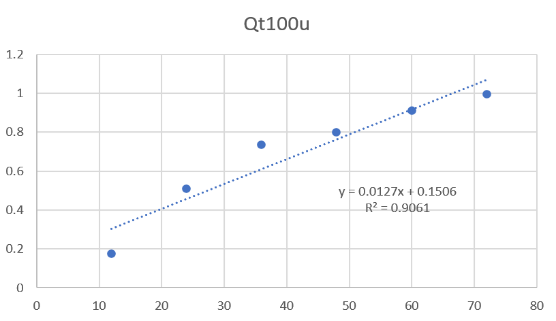 |
| Higuchi model  R^2^=0.963 | 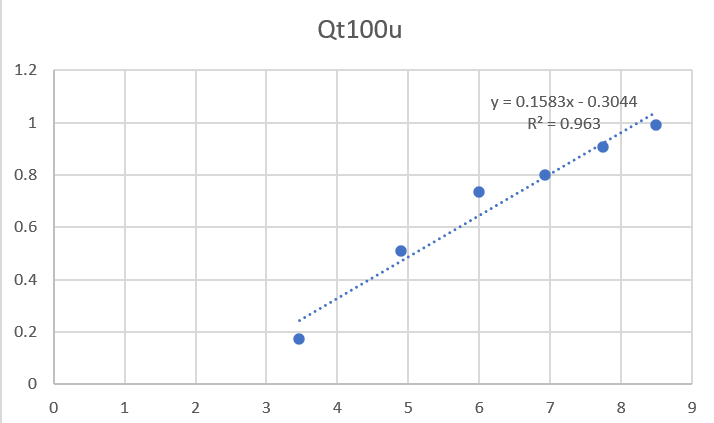 |
| First-order kinetic model  R^2^=0.8537 | 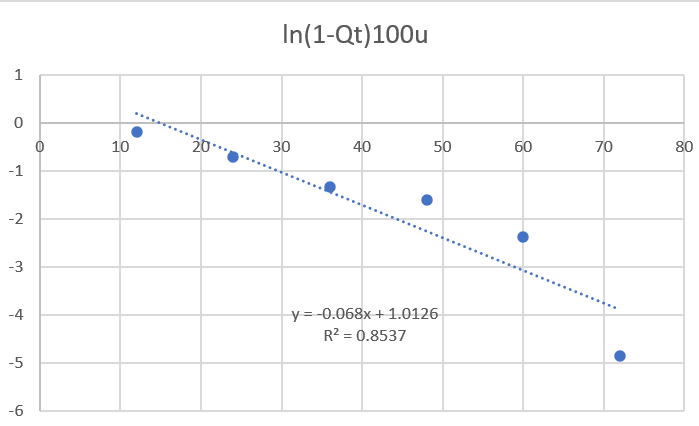 |
| Ritger-Peppas model  R^2^=0.9256 | 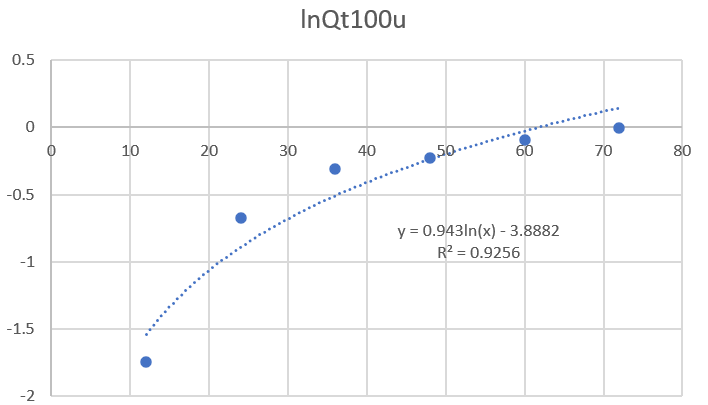 |

| **Gel10-Th150** | |
| --- | --- |
| Zero-order kinetic model  R^2^=0.9857 | 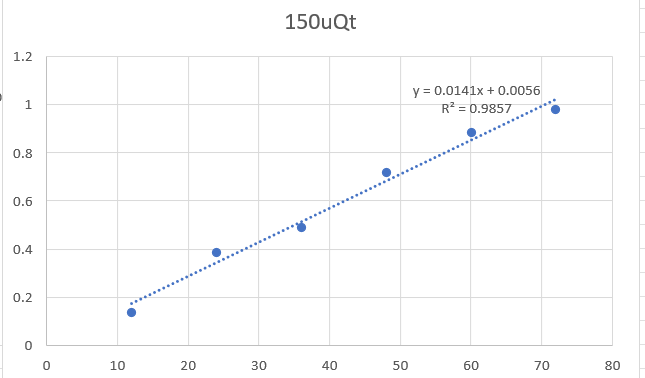 |
| Higuchi model  R^2^=0.9886 | 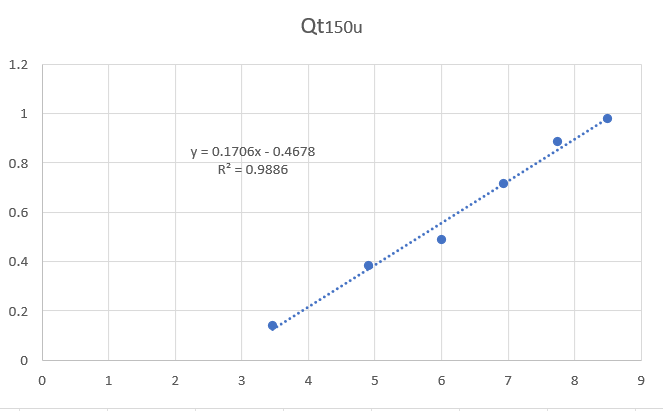 |
| First-order kinetic model  R^2^=0.8631 | 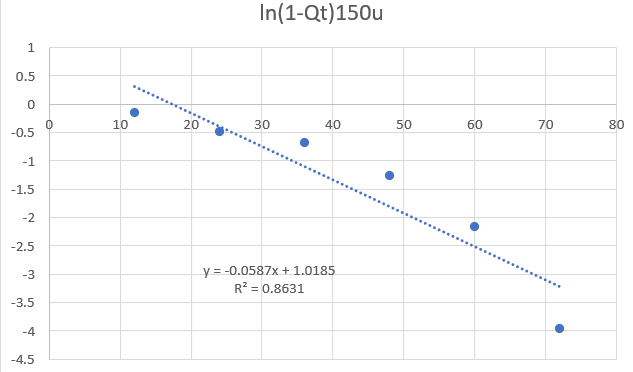 |
| Ritger-Peppas model  R^2^=0.9818 | 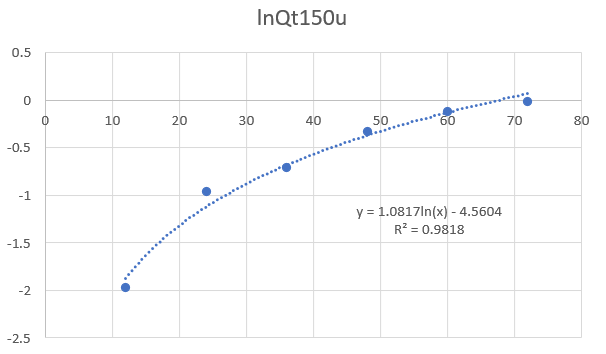 |

| **Gel10-Th200** | |
| --- | --- |
| Zero-order kinetic model  R^2^=0.9889 | 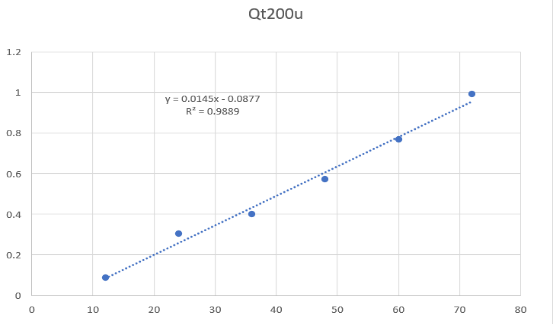 |
| Higuchi model  R^2^=0.9624 | 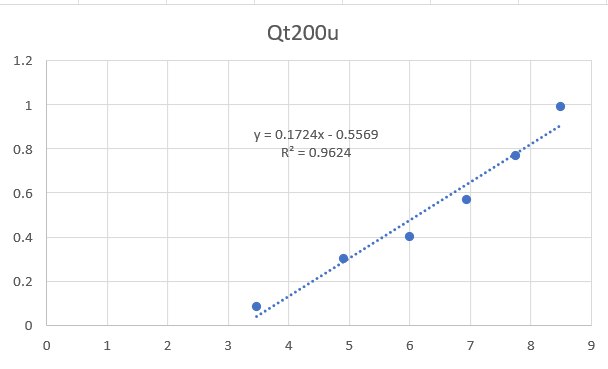 |
| First-order kinetic model  R^2^=0.6788 | 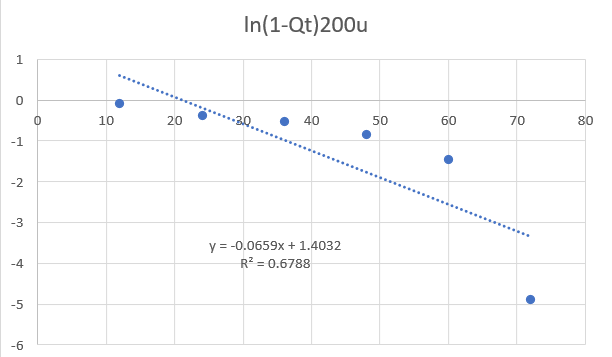 |
| Ritger-Peppas model  R^2^=0.9808 | 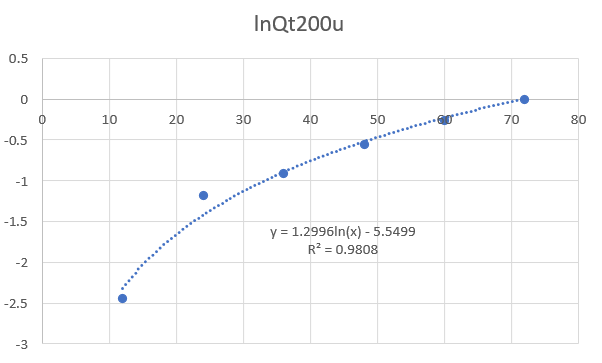 |

Tab. S1. Analysis of Kinetic Models for Drug Release.

Formulas for four drug release kinetics:

| Zero-order kinetic model | Q_(t)_=kt+Q_0_ |
| --- | --- |
| Higuchi model | Q_(t)_=kt^1/2^ |
| First-order kinetic model | ln(1−Q_(t)_)=−kt+ln(1−Q_0_​) |
| Ritger-Peppas model | lnQ_(t)_=lnk+nlnt |

The characteristics of these four kinetic models are as follows:

The core feature of the zero-order kinetic model is that the drug release rate is constant and independent of time, and the released amount has a linear relationship with time.

The core feature of the first-order kinetic model is that the drug release rate is proportional to the remaining drug amount, the released amount increases exponentially with time, with a faster release in the initial stage and a gradual slowdown afterward.

The core feature of the Higuchi model is that it is based on diffusion theory, applicable to the situation where drugs are released from porous matrices, and the released amount has a linear relationship with the square root of time.

The core feature of the Ritger-Peppas model is that it is a more universal empirical model, applicable to the initial stage of drug release from polymer matrices (usually with a released amount ≤ 60%), and can distinguish release mechanisms such as diffusion and erosion. Specifically, when n = 0.5, the release is controlled by pure diffusion; when 0.5 < n < 1, the release is controlled by both diffusion and matrix swelling; when n = 1, it is zero-order release (controlled by erosion).
